# A Global Assessment of Eye Health and Quality of Life: A Systematic Review of Systematic Reviews

**DOI:** 10.1001/jamaophthalmol.2021.0146

**Published:** 2021-02-12

**Authors:** Lama Assi, Fatimah Chamseddine, Perla Ibrahim, Hadi Sabbagh, Lori Rosman, Nathan Congdon, Jennifer Evans, Jacqueline Ramke, Hannah Kuper, Matthew J. Burton, Joshua R. Ehrlich, Bonnielin K. Swenor

**Affiliations:** 1 Wilmer Eye Institute, Johns Hopkins University School of Medicine, Baltimore, Maryland; 2 Clinical Research Institute, American University of Beirut Faculty of Medicine, Beirut, Lebanon; 3 Department of Ophthalmology, American University of Beirut Faculty of Medicine, Beirut, Lebanon; 4 Welch Medical Library, Johns Hopkins University School of Medicine, Baltimore, Maryland; 5 Centre for Public Health, Queen's University Belfast School of Medicine Dentistry and Biomedical Sciences, Belfast, United Kingdom; 6 Zhongshan Ophthalmic Center, Sun Yat-sen University, Guangzhou, China; 7 International Centre for Eye Health, London School of Hygiene & Tropical Medicine, London, United Kingdom; 8 School of Optometry and Vision Science, University of Auckland, Auckland, New Zealand; 9 International Centre for Evidence in Disability, London School of Hygiene & Tropical Medicine, London, United Kingdom; 10 Moorfields Eye Hospital, London, United Kingdom; 11 Department of Ophthalmology and Visual Sciences, University of Michigan, Ann Arbor; 12 Institute for Healthcare Policy and Innovation, University of Michigan, Ann Arbor; 13 Department of Epidemiology, Johns Hopkins Bloomberg School of Public Health, Baltimore, Maryland

## Abstract

**Importance:**

More than 1 billion people worldwide have vision impairment or blindness from potentially preventable or correctable causes. Quality of life, an important measure of physical, emotional, and social well-being, appears to be negatively associated with vision impairment, and increasingly, ophthalmic interventions are being assessed for their association with quality of life.

**Objective:**

To examine the association between vision impairment or eye disease and quality of life, and the outcome of ophthalmic interventions on quality of life globally and across the life span, through an umbrella review or systematic review of systematic reviews.

**Evidence Review:**

The electronic databases MEDLINE, Ovid, Embase, Cochrane Database of Systematic Reviews, Proquest Dissertations, and Theses Global were searched from inception through June 29, 2020, using a comprehensive search strategy. Systematic reviews addressing vision impairment, eye disease, or ophthalmic interventions and quantitatively or qualitatively assessing health-related, vision-related, or disease-specific quality of life were included. Article screening, quality appraisal, and data extraction were performed by 4 reviewers working independently and in duplicate. The Joanna Briggs Institute critical appraisal and data extraction forms for umbrella reviews were used.

**Findings:**

Nine systematic reviews evaluated the association between quality of life and vision impairment, age-related macular degeneration, glaucoma, diabetic retinopathy, or mendelian eye conditions (including retinitis pigmentosa). Of these, 5 were reviews of quantitative observational studies, 3 were reviews of qualitative studies, and 1 was a review of qualitative and quantitative studies. All found an association between vision impairment and lower quality of life. Sixty systematic reviews addressed at least 1 ophthalmic intervention in association with quality of life. Overall, 33 unique interventions were investigated, of which 25 were found to improve quality of life compared with baseline measurements or a group receiving no intervention. These interventions included timely cataract surgery, anti–vascular endothelial growth factor therapy for age-related macular degeneration, and macular edema.

**Conclusions and Relevance:**

There is a consistent association between vision impairment, eye diseases, and reduced quality of life. These findings support pursuing ophthalmic interventions, such as timely cataract surgery and anti–vascular endothelial growth factor therapy, for common retinal diseases, where indicated, to improve quality of life for millions of people globally each year.

## Key Points

**Question:**

What is the association between vision impairment, eye diseases, or ophthalmic interventions and quality of life?

**Findings:**

In this cross-sectional study, vision impairment and eye diseases were associated with lower quality of life. More than half of the ophthalmic interventions included had a positive association with quality of life.

**Meaning:**

The associations of quality of life with vision impairment and the improvements in quality of life with ophthalmic interventions support efforts to improve access to ophthalmic treatments globally to reach the millions of people affected by eye disease each year.

## Teaser

This cross-sectional study examines the association between vision impairment or eye disease and quality of life and the outcome of ophthalmic interventions on quality of life globally and across the life span.

## Introduction

At least 2.2 billion people worldwide have a vision impairment, of whom more than 1 billion have moderate or severe vision impairment or blindness from a preventable or potentially correctable cause, including refractive error, presbyopia, and cataract.^1^ Existing evidence suggests that vision impairment is associated with lower quality of life,^2^ defined as physical, emotional, and social well-being. Visual impairment is also linked to lower vision-related quality of life^3^ or daily visual function and the ability to perform visual tasks.

Over the past decade, quality-of-life measures have gained popularity in ophthalmology research, including clinical trials, as the value of patient-reported outcomes in measuring well-being and visual function is being recognized.^4^ However, to our knowledge, there has yet to be a global synthesis of the evidence about quality of life and eye health, despite the numerous systematic reviews about vision impairment, eye diseases, or ophthalmic interventions and quality of life.^5,6,7^

Therefore, the objective of this umbrella review, which is a systematic review of systematic reviews, is to examine the association between vision impairment or specific eye diseases and reduced quality of life, and the effectiveness that ophthalmic interventions can have on improving quality of life.

## Methods

This study forms part of the work for the forthcoming *Lancet Global Health* Commission on Global Eye Health.^8^ We followed the Preferred Reporting Items for Systematic Review and Meta-Analysis (PRISMA) reporting guidelines (eAppendix 1 in the Supplement). A protocol was published^9^ and registered on the Open Science Framework Registries (https://osf.io/qhv9g). Changes to the protocol are noted in eAppendix 2 in the Supplement.

A comprehensive search was performed using the electronic databases MEDLINE, Ovid, Embase, Cochrane Database of Systematic Reviews, Proquest Dissertations, and Theses Global from inception through June 29, 2020 (a sample search strategy is in the eFigure in the Supplement). OpenGrey, the Agency for Healthcare Research and Quality, and references of included reviews were searched for additional articles. Inclusion and exclusion criteria are listed in Table 1.

**Table 1.** Inclusion and Exclusion Criteria

Characteristic	Inclusion criteria	Exclusion criteria
Study type	Systematic reviews, defined as reviews that include all of these items: a research question, search strategy, the sources searched, inclusion/exclusion criteria, screening methods, assessment of included studies’ quality, and information about data synthesis	Reviews that did not meet the working definition of a systematic review; systematic reviews that included case series or expert opinion pieces
Population	Systematic reviews with participants with vision impairment or eye disease	Systematic reviews with participants with stroke-associated vision impairment, since these vision impairments and consequences (eg, visual neglect, visual hallucinations) are different from eye pathologies
Interventions	For systematic reviews of interventional studies, systematic reviews that assessed interventions that aim to improve or preserve vision, prevent or stop the progression of vision impairment, or improve vision function	For systematic reviews of interventional studies, systematic reviews that assessed psychologic interventions, such as coping strategies
Outcomes	Systematic reviews that reported on quality-of-life outcomes, such as health-related, vision-related, or disease-specific quality-of-life questionnaires, or qualitative assessments of physical, emotional, and social well-being and vision function in day-to-day life	Systematic reviews that reported on patient satisfaction and patient-reported symptoms
Other	None	Conference abstracts and articles not in English; systematic reviews that had an updated version available (they were used as an additional reference only if the updated review referred to them)

Study selection, quality appraisal, and data collection were performed by 4 reviewers (L.A., F.C., P.I., and H.S.) independently and in duplicate using Covidence software (Covidence Inc).^10^ Titles and abstracts were screened to identify potentially relevant articles. Full texts of these articles were assessed for eligibility based on the inclusion and exclusion criteria. Reviews that aimed to identify studies with quality-of-life outcomes but did not find any were excluded, since they had no results to be extracted.

Reviews underwent quality appraisal using the Joanna Briggs Institute Critical Appraisal Checklist for Systematic Reviews and Research Syntheses.^11^ Reviews for which any of the items “clear review question,” “appropriate inclusion criteria,” “appropriate search strategy,” or “appropriate criteria for critical appraisal” were graded as unclear or no were excluded. Data collection was performed using the Joanna Briggs Institute Data Extraction Form for Systematic Reviews and Research Syntheses.^12^

Results of the reviews on vision impairment or eye diseases and ophthalmic interventions were presented separately. Within the reviews on ophthalmic interventions, 2 types of comparisons were identified: (1) those that compared the quality of life of a group receiving an intervention with baseline quality of life in the same group or a control group receiving no intervention, a placebo, or sham therapy and (2) those that compared the quality of life of a group receiving 1 intervention with a group receiving another intervention, without comparison with a baseline or a group that received no intervention. Results from each type of comparison were described separately, and only comparisons with baseline or a group receiving no intervention were included in the Tables summarizing findings (Table 2 and Table 3).

**Table 2.** Findings on the Outcomes of Ophthalmic Interventions and Quality of Life by Systematic Review^a^

Source	Year	Country of lead author	Quality of life outcome or measure	Assessment tools	Assessment	Participants, No.^b^	Studies, No.	Study Type	Countries of primary studies^c^	Findings	Quality of evidence
**Age-related cataract**											
Chou, et al^13^	2016	UK	VRQOL after cataract surgery	NR	Treatment of early impairment in visual acuity due to cataract error	1627	5	Cohort studies	NR	Three studies found moderate improvement in VRQOL and function after cataract surgery. Two studies found similar VRQOL in the groups with vs without cataract surgery (measures of association not provided).	Fair
Hodge, et al^14^	2007	Canada	Patient QOL and satisfaction	NR	Expedited (<6 wk waiting time) first-eye or second-eye cataract surgery vs control group awaiting surgery	NR	2	RCTs	The UK	Better QOL in the group that received cataract surgery in <6 wk vs a group awaiting surgery (routine waiting time, >6 mo) at 6 mo after randomization (measures of association not provided).	NR
Conner-Spady, et al^15^	2007	Canada	VRQOL	VF-14	Expedited (<6 wk waiting time) first-eye or second-eye cataract surgery vs control group awaiting surgery	514	2	RCTs	The UK	Significant VRQOL benefits in the groups that received cataract surgery vs control groups still awaiting cataract surgery 6 mo after randomization. Deterioration in VRQOL in the control group while waiting (measures of association not provided).	NR
Casparis, et al^16^	2017	Switzerland	VRQOL	Impact of Vision Impairment questionnaire	Immediate vs no or delayed cataract surgery (>6 mo) among patients with AMD	56	1	RCT	Australia	Better VRQOL in the immediate-cataract surgery group vs the control group awaiting surgery (waiting time >6 mo) after 6 mo of follow-up (MD, 1.60 [95% CI, 0.61-2.59]; *I*^2^ = NA).	Low
Ishikawa, et al^17^	2013	Canada	Self-reported visual functioning	VF-14, Tailored questionnaire, or ADVS	Second-eye cataract surgery vs cataract surgery in 1 eye only	1554	7	3 RCTs, 4 cohort studies	NR	Better VRQOL after second-eye cataract surgery vs cataract surgery in 1 eye only was reported in all studies. Four studies found that the magnitude of improvement was smaller after second-eye surgery than after first-eye surgery (measures of association not provided).	Moderate
Frampton, et al^18^	2014	UK	VRQOL	VF-14	Second-eye cataract surgery vs cataract surgery in 1 eye only	535	2	RCT	The UK and Spain	Better VRQOL existed in those post–expedited second-eye surgery vs those awaiting second-eye cataract surgery, but the difference was not clinically meaningful (VF-14: study 1: MD, 7.5 [95% CI, 5.1-9.9]; *P* < .001; study 2: MD, 8.24 [95% CI, 4.35-12.36]; *P* < .001).	NR
Ishikawa, et al^17^	2013	Canada	HRQOL	SIP, HRQOL, SF-12, SF-36, EQ-5D, or SRS	Second-eye cataract surgery vs cataract surgery in 1 eye only	1261	6	3 RCTs, 3 cohort studies	NR	Inconsistent and mixed results. One study found improvement in HRQOL after second-eye cataract surgery, 3 found no improvement in HRQOL, and 2 found mixed results (measures of association not provided).	NR
Frampton, et al^18^	2014	UK	HRQOL	SF-12, SF-36, or EQ-5D	Second-eye cataract surgery vs cataract surgery in 1 eye only	743	3	RCT	The UK and Spain	Mixed results; 2 studies found no significant improvement in HRQOL after second-eye cataract surgery. One study found clinically relevant improvement in the mental health component of HRQOL after expedited vs no second-eye surgery (point difference, 1.90 [95% CI, 0.03-3.79]; *P* < .05).	NR
Riaz, et al^19^	2009	UK	VRQOL	NR	Extracapsular cataract extraction with posterior chamber IOL vs intracapsular cataract extraction with aphakic glasses	3400	1	RCT	India	Improved VRQOL in both groups, with the advantage of extracapsular cataract extraction with posterior chamber IOL vs aphakic glasses across all categories (measures of association not provided).	NR
**Refractive error**											
Chou, et al^13^	2016	UK	VRQOL	NEI-VFQ	Treatment of early impairment in visual acuity due to uncorrected refractive error	282	2	RCT	NR	Beneficial effects of corrective lenses on VRQOL or vision-related function in the group with immediate correction of refractive error with eyeglasses compared with delayed treatment (scores on the NEI-VFQ were improved by a mean of approximately 10 of 100 points in the immediate-treatment groups).	Fair
**Age-related macular degeneration**											
Chou, et al^13^	2016	UK	VRQOL	NR	Anti-VEGF injections vs control for neovascular AMD	NR	3	Trials??	NR	Mild to moderate improvements in VRQOL in the groups who took anti-VEGF vs sham injections, but the differences were not always significant (measures of association not provided).	Fair
Solomon, et al^20^	2019	UK	VRQOL	NEI-VFQ	Anti-VEGF injections vs control for neovascular AMD	1134	2	RCT	The US, France, Germany, Hungary, Czech Republic, and Australia	Greater improvement in VRQOL in the ranibizumab than control groups (no anti-VEGF) after 1 y of follow-up (MD, 6.7 [95% CI, 3.4-10.0]; *I*^2^ = 68.3%).	Moderate
Sarwar, et al^21^	2016	US	VRQOL	NEI-VFQ-25	Aflibercept or ranibizumab therapy vs baseline for AMD	2412	2	RCT	The US, Canada, Argentina, Australia, Austria, Brazil, Belgium, Colombia, Czech Republic, France, Germany, Hungary, India, Israel, Italy, Japan, Latvia, Mexico, the Netherlands, Poland, Portugal, South Korea, Singapore, Slovakia, Spain, Sweden, Switzerland, and the UK	Improvement in VRQOL from baseline to 1 y in both aflibercept and ranibizumab groups to a similar extent (MD, −0.39 [95% CI, −1.71 to 0.93]; *I*^2^ = 54.71%).	High
Giansanti, et al^22^	2009	Italy	VRQOL	NEI-VFQ-25	Macular/submacular surgery vs observation for subfoveal neovascular AMD	689	2	RCT	The US	Better VRQOL in the surgery vs observation group at 1 y (RR, 1.35 [95% CI, 1.09-1.68]; *I*^2^ = 0.0%).	Low
Evans, et al^23^	2010	UK	VRQOL	Daily Living Tasks Dependent on Vision questionnaire	Radiotherapy vs observation for neovascular AMD	203	1	RCT	The UK	No differences in VRQOL between treatment and observation groups 12 or 24 mo after treatment (measures of association not provided).	NR
Evans, et al^23^	2017	UK	VRQOL	NEI-VFQ-25	Multivitamin supplements vs placebo or no treatment for AMD	110	1	RCT	Italy	Better VRQOL in the multivitamin supplements group vs placebo at 24 mo (MD, 12.3 [95% CI, 4.24-20.36]).	Low
Evans, et al^23^	2017	UK	QOL	NEI VFQ-25	Lutein and/or zeaxanthin vs placebo for AMD	108	1	RCT	China	Similar VRQOL changes in the intervention and placebo groups at 12 mo (MD, 1.48 [95% CI, −5.53 to 8.49]).	Low
Liu, et al^24^	2014	China	VRQOL	VFQ	Lutein and/or zeaxanthin vs placebo for AMD	253	2	RCT	NR	No significant difference in VRQOL improvement between groups (weighted MD, 6.51 [95% CI, −6.16 to 19.17]).	NR
**Retina (other)**											
Virgili, et al^25^	2018	Italy	VRQOL	NEI-VFQ-25	Anti-VEGF therapy vs laser photocoagulation for diabetic macular edema	412	3	RCT	Canada, Europe, Australia, Canada, and Turkey	Improvement in VRQOL from baseline to 6 or 12 mo in both groups; greater improvement in the ranibizumab group vs laser photocoagulation group (mean change in composite score, 5.14 [95% CI, 2.96-7.32]).	Moderate
Braithwaite, et al^26^	2014	UK	VRQOL	NEI-VFQ-25	Anti-VEGF injection vs sham injection for macular edema secondary to central retinal vein occlusion	743	3	RCT	Argentina, Asia/Pacific, Canada, Colombia, Europe, India, Israel, and the US	Significant improvement in VRQOL in the anti-VEGF vs sham groups at 6 mo (MD not provided; range, 6.2 to 7.5, based on 1 study).	Moderate
Zhou, et al^27^	2014	China	VRQOL	NEI-VFQ-25	Anti-VEGF injection vs sham injection for macular edema secondary to central retinal vein occlusion	743	3	RCT	Argentina, Asia/Pacific, Canada, Colombia, Europe, India, Israel, and the US	Significant improvement in VRQOL in the anti-VEGF vs sham groups at 6 mo (MD, 4.58 [95% CI, 2.93-6.23]; *P* < .001; *I*^2^ = 0%).	High
Ford, et al^28^	2014	UK	VRQOL	NEI-VFQ-25	Anti-VEGF injection vs sham injection for macular edema secondary to central retinal vein occlusion	782	3	RCT	NR	Significantly better changes in VRQOL in both the aflibercept and ranibizumab groups vs sham groups at 6 mo (MDs, 6.4, 4.0, nad 3.4, respectively; confidence intervals not provided).	NR
Mitry, et al^29^	2013	UK	VRQOL	NEI-VFQ-25	Anti-VEGF injection vs sham injection for macular edema secondary to branch retinal vein occlusion	397	1	RCT	The US	Greater VRQOL improvement in the ranibizumab groups vs sham group at 6 mo of treatment (change in NEI-VFQ-25 composite score, 9.3 [95% CI, 7.2-11.4] in the 0.3-mg ranibizumab group; 10.4 [95% CI, 8.3-12.4] in the 0.5-mg ranibizumab group; and 5.4 [95% CI, 3.6-7.3] in the sham group; *P* < .005 for each group vs the sham group).	NR
Zhu, et al^30^	2016	China	VRQOL	NEI-VFQ-25	Anti-VEGF injection vs sham injection for choroidal neovascularization secondary to pathological myopia	121	1	RCT	Hong Kong, Japan, Korea, Singapore, and Taiwan	Better VRQOL outcomes in the anti-VEGF vs sham groups (mean change in NEI-VFQ-25 score, 5.72 [95% CI, 1.60-9.84].	Moderate
Lescrauwaet, et al^31^	2019	Belgium	VRQOL	NEI-VFQ-25	Ocriplasmin injection vs sham or placebo injection for symptomatic vitreomacular traction	870	2	RCT	NR	A higher proportion of people in the ocriplasmin group had a clinically meaningful improvement in VRQOL vs those in the control group (difference in proportions, 11.8% [95% CI, 3.8%-19.7%]; *P* = .004).	NR
Neffendorf, et al^32^	2017	UK	VRQOL	VFQ	Ocriplasmin injection vs sham or placebo injection for symptomatic vitreomacular adhesion	656	2	RCT	The US, Belgium, Czech Republic, Germany, Poland, Spain, and the UK	Greater improvement in VRQOL in the ocriplasmin group vs sham/placebo group at 6 mo (MD in improvement, 2.7 [95% CI, 0.8-4.6] points).	Moderate
Brito-García, et al^33^	2017	Spain	VRQOL	VAQ and VF-14	Nutritional supplementation treatments for hereditary retinal dystrophies (retinitis pigmentosa, Best disease)	52	2	RCT	The US and Canada	No significant differences in VRQOL were noted between the groups of participants with retinitis pigmentosa and Best disease who received nutritional supplementation vs those who did not.	NR
**Glaucoma**											
Rolim de Moura, et al^34^	2007	Brazil	VRQOL	NEI VFQ-25	Laser trabeculoplasty and topical β-blocker vs placebo for early open-angle glaucoma	255	1	RCT	NR	No significant difference in VRQOL between the treatment and placebo groups at 3 y.	NR
Chi, et al^35^	2020	Taiwan	QOL	GQL-15	Selective laser trabeculoplasty and medication or medication only for open-angle glaucoma	41	1	RCT	Unspecified countries in Asia	No significant change in QOL from baseline to follow-up at 6 mo in the selective laser trabeculoplasty and medication and medication-only groups.	NR
**Low vision**											
van Nispen, et al^36^	2020	the Netherlands	VRQOL	NEI-VFQ- 25, VA-LV-VFQ48, Activity Inventory, IVI	Vision rehabilitation using methods of enhancing vision (eg, low-vision outpatient service, customized prism glasses) vs passive control for adults with vision impairment	262	5	RCT	The US, Germany, and Canada	Small benefit in VRQOL favoring low vision rehabilitation, but the effects were moderately heterogenous and imprecisely estimated, including no benefit (standardized MD, −0.19 [95% CI, −0.54 to 0.15]; *I*^2^ = 34%).	Very low
van Nispen, et al^36^	2020	The Netherlands	HRQOL	EQ-5D, SF-36	Multidisciplinary vision rehabilitation (eg, low-vision rehabilitation plus home visit) vs passive control for adults with vision impairment	183	2	RCT	The UK and the US	Rehabilitation resulted in more favorable HRQOL, but estimates were very imprecise and included no effect (standardized MD, −0.08 [95% CI, −0.37 to 0.21]; *I*^2^ = 0%).	Very low
van Nispen, et al^36^	2020	The Netherlands	VRQOL	NEI- VFQ-25, VFQ-48 questionnaire	Multidisciplinary vision rehabilitation (eg, low-vision rehabilitation plus home visit) vs passive control for adults with vision impairment	193	2	RCT	The UK and the US	Both studies found better VRQOL with rehabilitation, but the effect was large in a large trial delivering intensive rehabilitation (standardized MD, 1.64 [95% CI, −2.05 to −1.24]) and small in the other study (standardized MD, −0.42 [95% CI, −0.90 to 0.07]).	Very low
**Vision screening**											
Evans, et al^37^	2018	UK	VRQOL	NEI- RQL-42	School vision screening and ready-made spectacles vs vision screening and custom-made spectacles	188	1	RCT	China	Improvement in VRQOL to a similar extent in both groups after wearing spectacles for 2 mo (change in NEI-RQL-42 score in the ready-made spectacles group, 4.65 [95% CI, 2.45-6.86]; similar change in the custom-made spectacles group).	Moderate
**Rhinoconjunctivitis**											
Erekosima, et al^38^	2014	UK	QOL	RQOL questionnaire	Subcutaneous immunotherapy vs placebo for rhinoconjunctivitis	539	4	RCT	NR	Greater improvement in disease-specific QOL among adults in the subcutaneous immunotherapy group vs placebo group (measures of association not provided).	High
Kim, et al^39^	2013	US	QOL	RQOL questionnaire	Subcutaneous immunotherapy vs placebo for rhinoconjunctivitis	350	2	RCT	NR	Significant improvement in disease-specific QOL in the subcutaneous immunotherapy arm vs the control group (measures of association not provided).	Low
Kim, et al^39^	2013	US	QOL	Pediatric and Adolescent RQOL questionnaires	Sublingual immunotherapy vs placebo for rhinoconjunctivitis among children only	461	2	RCT	NR	No improvement in disease-specific QOL in the sublingual immunotherapy group vs placebo among children.	NR
Lin, et al^40^	2013	US	Disease-specific quality of life for rhinoconjunctivitis and asthma	RQOL questionnaire	Sublingual immunotherapy vs placebo for rhinoconjunctivitis among children and adults	819	8	RCT	NR	Improvement in disease-specific QOL in the sublingual immunotherapy group in 7 of 8 studies. Results were statistically significant in 4 of the studies, and a strong magnitude of association (>40% difference in effect) was reported in 2 studies.	Moderate
Rodrigo, et al^41^	2010	Uruguay	QOL	RQOL Questionnaire	Intranasal fluticasone furoate vs placebo for seasonal allergic rhinitis	2219	5	RCT	NR	Significant improvement in disease-specific QOL in the intranasal fluticasone furoate group vs placebo (weighted MD, −0.68 [95% CI, −0.80 to −0.56]; *I*^2^ = 0%).	NR
Rodrigo, et al^41^	2010	Uruguay	QOL	RQOL Questionnaire	Intranasal fluticasone furoate vs placebo for perennial allergic rhinitis	919	3	RCT	NR	Significant improvement in disease-specific QOL in the intranasal fluticasone furoate group vs placebo (weighted MD, −0.51 [95% CI, −0.76 to −0.22]; *I*^2^ = 44%).	NR
**Uveitis**											
Urruti-coechea-Arana, et al^42^	2019	Spain	VRQOL	NEI-VFQ-25	Adalimumab or dexamethasone vs placebo in the treatment of uveitis	443	2	RCT	NR	Significantly greater improvement in VRQOL in the adalimumab group vs placebo among participants with active uveitis (measures of association not provided). However, no differences were found in the treatment group vs placebo among those with inactive uveitis.	NR
Squires, et al^43^	2017	UK	VRQOL	NEI-VFQ-25	Adalimumab or dexamethasone vs placebo in the treatment of uveitis	681	3	RCT	Europe, North America, and Australia	Significantly greater improvement in VRQOL in the adalimumab vs placebo group in patients with active uveitis (MD, 4.20 [95% CI, 1.02-7.38]; *P* = .01), but not in those with inactive uveitis (MD, 2.12 [95% CI, −0.84 to 5.08]; *P* = .16). Similarly, significant VRQOL benefits noted using dexamethasone implant vs sham procedure.	NR
Squires, et al^43^	2017	UK	HRQOL	EQ-5D	Adalimumab or dexamethasone vs placebo in the treatment of uveitis	452	2	RCT	Europe, North America, and Australia	Significantly greater improvement in HRQOL in the adalimumab vs placebo groups among participants with active uveitis (MD, 0.04 [95% CI, 0.00-0.07]). However, no differences were found in the treatment group vs placebo among those with inactive uveitis (MD, 0.00 [95% CI, –0.03 to 0.04]).	NR
**Trichiasis**											
Burton, et al^44^	2015	UK	VRQOL	??	Perioperative azithromycin vs no azithromycin	1903	2	RCT	Gambia and Ethiopia	VRQOL (vision function, eye comfort, and physical functioning) improved following surgery; however, studies did not analyze by groups allocated to azithromycin vs control (measures of association not provided).	NR
**Thyroid eye disease or Graves ophthalmopathy**											
Viani, et al^45^	2012	Brazil	QOL	NR	Radiotherapy vs sham radiotherapy for thyroid eye disease	88	1	RCT	NR	No differences in QOL in the radiotherapy vs sham radiotherapy groups.	NR
Rajendram, et al^46^	2012	UK	QOL	Graves Ophthalmopathy QOL, Euro-QoL, Sickness Impact Profile, and Medical Outcomes Study Short-General Health Survey questionnaires	Radiotherapy vs sham radiotherapy for thyroid eye disease	88	1	RCT	NR	No significant differences in QOL between the radiotherapy vs sham radiotherapy groups.	NR

Abbreviations: anti-VEGF, anti–vascular endothelial growth factor; EQ-5D, EuroQol-5 Dimension; GQL-15, Glaucoma Quality of Life-15; HRQOL, health-related quality of life; IVI, impact of vision impairment; MD, mean difference; NEI-RQL-42, National Eye Institute Refractive Error Quality of Life Instrument–42; NEI-VFQ-25, National Eye Institute 25-Item Visual Function Questionnaire; NR, not reported; QOL, quality of life; RCT, randomized clinical trial; RR, risk ratio; RQOL, rhinoconjunctivitis quality of life; SF-12, 12-Item Short Form Survey; SF-36, 36-Item Short Form Health Survey; SIP, Sickness Impact Profile; VRQOL, vision-related quality of life.

^a^ List of included studies in eTable 4 in the Supplement.

^b^ Informing the specific outcome (a given systematic review may have included more studies for other outcomes).

^c^ Indicates overlap of studies. In this column, we report the name of the study or first author and year of publication (as reported by the systematic review). Only applicable for outcomes assessed by more than 1 systematic review.

**Table 3.** Findings on the Association Between Vision Impairment or Eye Disease and Quality of Life by Systematic Review^a^

Systematic review^a^	Year	Country of lead author	Quality of life outcome or measure	Assessment instruments	Participants, No.^b^	Participant type	Studies, No.	Study type	Countries of primary studies^b^	Results/findings
**Systematic reviews of quantitative studies**									
Nyman, et al^47^	2010	UK	Mental health subscale in QOL questionnaires	NEI-VFQ-25 and SF-36	33 648	Working-age adults (18-59 y)	11	NR	The US, Korea, and India	Vision impairment was associated with modestly lower mental health scores on the NEI-VFQ-25 than control participants (average mean difference, 14.5% [range, 0.6%-35%]). The SF-36 was less sensitive than the NEI-VFQ-25 in detecting lower mental health among those with vision impairment vs controls (mean difference, 3%; 1 study only).
Nyman, et al^47^	2010	UK	QOL	NR	2622	Working-age adults (18-59 y)	3	NR	The US, Sweden, and Australia	Vision impairment was associated with lower QOL across a range of measures, such as higher odds of reporting “not feeling full of life” vs control participants (OR, 4.63 [95% CI, 2.1-9.8])
Tseng, et al^49^	2018	Taiwan	QOL	WHOQOL-BREF, CDCHRQOL, SF-36, IVI, NHVQOL, EQ-5D, NEI-VFQ-25, and WHO/PBD VF20	NR	Older adults	15	14 cross-sectional and 1 longitudinal study	Australia, Canada, Europe, Germany, Korea, Nepal, New Zealand, Nigeria, Philippines, Taiwan, and the US	Vision impairment was associated with lower QOL in 14 of 15 studies (eg, OR, 2.20 [95% CI, 1.10-4.90] in 1 study examining vision impairment and generic health-related QOL; in another study examining vision impairment among older adults, the difference in SF-36 scores was 6.7 (95% CI, 3.37-10.1; *P* < .001). An increase in vision impairment severity was associated with lower QOL (eg, β, 0.31 [95% CI, 0.11-0.52]; *P* = .003).
Schakel, et al^48^	2019	The Netherlands	Fatigue severity	SF-36 vitality subscale or Fatigue Assessment Scale	10 870	Adults	14	7 case-control and 7 cross-sectional studies	Australia, Brazil, China, Greece, Japan, Nepal, the Netherlands, Taiwan, and the US	Vision impairment was associated with higher levels of fatigue in affected participants vs control participants with normal sight (standardized mean difference [SMD], −0.36 [95% CI, −0.50 to −0.22]; *I*^2^ = 84%).
Schakel, et al^48^	2019	The Netherlands	Fatigue odds	SF-36 vitality subscale or Fatigue Assessment Scale	8053	Adults	4	2 cross-sectional and 2 case-control studies	Canada, Czech Republic, Germany, and the Netherlands	Vision impairment was associated with higher odds of fatigue in affected participants vs control participants with normally sight (pooled adjusted OR, 2.61 [95% CI, 1.69-4.04]; *I*^2^ = 90%).
Wang, et al^50^	2017	China	Disease-specific QOL	GQL-15 questionnaire	253	Individuals with glaucoma	2	NR	Australia and Nigeria	Glaucoma was associated with significantly higher (ie, poorer) QOL summary scores (SMD , 0.94 [95% CI, 0.73-1.16]; *P* < .001; *I*^2^ = 0%), and subscale scores for all 4 factors (central and near vision: SMD, 0.82 (95% CI, 0.61-1.04]; *P* < .001; peripheral vision: SMD, 0.74 [95% CI, 0.53-0.96]; *P* < .001; dark adaption and glare: SMD, 1.02 [95% CI, 0.80-1.24]; *P* < .001; outdoor mobility: SMD, 0.60 [95% CI, 0.39-0.81]; *P* < .001) vs control group without glaucoma.
Khoo, et al^6^	2019	Singapore	Psychosocial functioning	Depression or anxiety questionnaires and mental health QOL subscale scores	NR	Individuals with diabetic retinopathy	28	Cross-sectional	NR	Diabetic retinopathy was significantly associated with poor psychosocial functioning in 20 of 28 observational studies (measures of association not provided).
D’Amanda, et al^53^	2020	US	QOL	NEI-VFQ-25, VF-14, SF-12, HRQOL-14, WHOQOL-BREF, CHQ, SF-36, and PedsQL	905	Individuals with mendelian eye conditions	11	Cross-sectional	Brazil, Canada, France, Greece, Israel, Korea, the Netherlands, and the UK	Retinitis pigmentosa, Usher syndrome, mixed retinal dystrophies, and retinoblastoma (in children) were associated with lower overall QOL in 5 studies. CHARGE syndrome, albinism, retinoblastoma, and mixed retinal dystrophies were associated with lower QOL on certain subscales (not specified) in 6 studies (measures of association not provided).
**Systematic reviews of qualitative studies**									
Nyman, et al^7^	2012	UK	Emotional well-being	NA	NR	Older adults	NR	Qualitative	NR	Diagnosis of vision impairment was identified as a traumatic event in 8 studies. An array of emotions was reported around the time of diagnosis, including feelings of shock, fear, panic, distress, helplessness, and frustration.
Nyman, et al^7^	2012	UK	General functioning	NA	NR	Older adults	NR	Qualitative	NR	Vision impairment had a dramatic outcome on individuals’ daily lives and functioning. People reported having to relinquish independence (11 studies) and leisure pursuits (8 studies).
Bennion, et al^51^	2012	UK	Emotional well-being	NA	121	Individuals with age-related macular degeneration	5	Qualitative	NR	The diagnosis of age-related macular degeneration was described as a shocking even by participants. Some accepted the diagnosis, while others felt powerless and in despair. Negative thoughts and depression symptoms were not confined to those with the most severe cases.
D’Amanda, et al^53^^c^	2020	US	General and visual functioning	NA	430	Individuals with mendelian eye conditions	9^c^	Qualitative	Australia, the Netherlands, Ireland, Sweden, Tanzania, the UK, the US, and Zimbabwe	Vision impairment had a considerable association with visual and global daily functioning and specific aspects of life, such as education, employment, and relationships among people with mendelian eye conditions (albinism, mixed retinal dystrophies, Leber hereditary optic neuropathy, retinitis pigmentosa, Alstrom syndrome, and retinoblastoma).
Garip, et al^52^	2019	UK	Emotional well-being	NA	223	Individuals with retinitis pigmentosa	10^c^	Qualitative	Australia, the US, Republic of Korea, Ireland, the Netherlands, and the UK	The diagnosis of retinitis pigmentosa was commonly accompanied by shock, negative emotional states, and a loss of confidence. Participants reported fatigue, fear, isolation, and vulnerability as they coped with the disease and dealt with their own judgements and perceived stigma.
Garip, et al^52^	2019	UK	Visual functioning	NA	223	Individuals with retinitis pigmentosa	10^c^	Qualitative	Australia, the US, Republic of Korea, Ireland, the Netherlands, and the UK	The diagnosis of retinitis pigmentosa was accompanied by loss of visual acuity, hobbies, pastimes, and social support. Participants reported difficulty performing day-to-day tasks, such as reading, seeing in changing light conditions, shopping, driving, playing sports, taking part in leisure activities, and doing household chores.

Abbreviations: CDCHRQOL, Centers for Disease Control and Prevention Health-Related Quality of Life; CHARGE syndrome, coloboma, heart defects, atresia choanae, growth retardation, genital abnormalities, and ear abnormalities; CHQ, Children’s Health Questionnaire; EQ-5D, EuroQoL–5 Dimension; GQL-15 questionnaire, Glaucoma Quality of Life–15 questionnaire; HRQOL-14, Health-Related Quality of Life–14; IVI, impact of vision impairment; NA, not applicable; NEI-VFQ-25, National Eye Institute 25-Item Visual Function Questionnaire; NHVQoL, Nursing Home Vision-Targeted Health-Related Quality of Life; NR, not reported; OR, odds ratio; PedsQL, Pediatric Quality of Life Inventory; QOL, quality of life; SF-12, 12-Item Short Form Survey; SF-36, 36-Item Short Form Health Survey; VF-14, Visual Function Index; WHOQOL-BREF, World Health Organization Quality of Life BREF; WHO/PBD VF20, World Health Organization Prevention of Blindness and Deafness Visual Function–20.

^a^ Quality of evidence ratings were not reported for any study.

^b^ Informing the specific outcome (a given systematic review may have included more studies for other outcomes).

^c^ Two identical primary studies were included in both reviews.

When the same intervention or outcome was assessed by more than 1 review, the primary studies used by the reviews to inform the results were compared to assess the extent to which individual studies were included in more than 1 review. Results about associations were based on the reviews’ interpretation of the estimates and accompanied by the measure of association and quality of the evidence assessment when available in the published review. Overall findings were presented in Table 2 and Table 3.

## Results

As described in the PRISMA flowchart (eTable 1 in the Supplement), 8070 unique titles and abstracts were screened; of these, 685 relevant full-text articles were assessed for eligibility. Ten eligible systematic reviews addressed quality of life and vision impairment or eye diseases, and 205 assessed ophthalmic interventions. Of the reviews concerning ophthalmic interventions, 143 were excluded because they did not identify any eligible studies with quality-of-life data in the literature.

Results of the quality assessment are presented in eTable 2 in the Supplement. Three reviews were excluded, 2 for not having appropriate critical appraisal criteria, and 1 for not having appropriate inclusion criteria. This left 9 reviews on vision impairment or eye diseases and 60 reviews on ophthalmic interventions included in the current analysis. Review characteristics are summarized in eTable 2 in the Supplement.

### Vision Impairment and Eye Diseases

In total, 9 systematic reviews^6,7,47,48,49,50,51,52,53^ published between 2010 and 2020 evaluated the association between vision impairment or eye disease and quality of life. Four of them had corresponding authors in the UK^7,47,51,52^; the rest were in the US,^53^ Netherlands,^48^ Taiwan,^49^ China,^50^ and Singapore.^6^ Five^6,47,48,49,50^ were systematic reviews of observational quantitative studies, 3^7,51,52^ were reviews of qualitative studies, and 1^53^ was a review of both quantitative and qualitative studies (Table 3). None of the reviews graded the quality of the evidence.

The systematic reviews of observational quantitative studies focused on people with vision impairment, including adults^48^ and specifically working-age^47^ and older adults,^49^ people with glaucoma,^50^ diabetic retinopathy,^6^ and children and adults with mendelian eye conditions, including retinitis pigmentosa, Usher syndrome, and mixed retinal dystrophies.^53^ Among all the populations examined, vision impairment or eye diseases were associated with lower quality of life, including vision-related and health-related^49^ and glaucoma-specific^50^ quality of life. Moreover, people with vision impairment had poorer scores on quality-of-life subscales, such as mental health,^47^ psychosocial functioning,^6^ and fatigue (odds ratio, 2.61 [95% CI, 1.69-4.04]).^48^

The systematic reviews of qualitative studies assessed emotional well-being and daily functioning among older adults with vision impairment^7^ and age-related macular degeneration (AMD)^51^ and children and adults with mendelian eye conditions,^53^ including retinitis pigmentosa specifically in a second review.^52^ The 2 reviews^52,53^ that addressed retinitis pigmentosa included 2 overlapping primary studies. Emotional well-being among people with vision impairment, AMD, and retinitis pigmentosa was especially affected at the initial diagnosis, which was described as a shocking or traumatic event in the 3 reviews.^7,51,52^ Moreover, coping with AMD and retinitis pigmentosa was associated with negative thoughts, including depressive symptoms, fatigue, and isolation.^51,52^ Vision impairment and mendelian eye conditions specifically also affected general daily functioning; people reported having to relinquish their independence and giving up on leisure activities.^7,52,53^ Difficulties performing visual tasks, such as reading and seeing in changing light conditions, were also reported by people with retinitis pigmentosa.^52^

Overall, 5 exposures (vision impairment; AMD; diabetic retinopathy; mendelian eye conditions, including retinitis pigmentosa; and glaucoma) were evaluated for their association with quality of life. A summary of findings is presented in the Box.

Box. **Summary of Findings From Systematic Reviews of Vision Impairment or Eye Disease Associated With Lower Quality of Life**

**Vision Impairment**

- Vitality subscale (fatigue) of health-related quality of life among adults with vision impairment^48^
- Mental health subscale of health-related and vision-related quality of life among working-age adults with vision impairment^47^
- Vision-related and health-related quality of life among older adults with vision impairment^49^
- Emotional well-being and general functioning among older adults with vision impairment^7^

**Age-Related Macular Degeneration**

- Emotional well-being among people with age-related macular degeneration^51^

**Diabetic Retinopathy**

- Psychosocial functioning among people with diabetic retinopathy^6^

**Mendelian Eye Conditions**

- Emotional well-being among people with retinitis pigmentosa^52^
- General and visual functioning among people with mendelian eye conditions, including retinitis pigmentosa^53^

**Glaucoma**

- Glaucoma-specific quality of life among people with glaucoma^50^

### Ophthalmic Interventions

In total, 60 systematic reviews published between 2005 and 2020 evaluated ophthalmic interventions using quality-of-life outcomes. Seventeen had corresponding authors in the UK,^18,19,23,26,28,29,32,37,42,43,44,46,54,55,56,57,58,59^ 13 in the US,^13,20,21,38,39,40,60,61,62,63,64,65,66^ 6 in China,^24,27,30,67,68,69^ 6 in Italy,^22,25,70,71,72,73^ 3 in Brazil,^34,45,74^ 3 in Canada,^14,15,17^ 2 in Denmark,^75,76^ 2 in Spain,^33,42^ and 1 each in Uruguay,^41^ Switzerland,^16^ Bahrain,^73^ Germany,^77^ Belgium,^31^ the Netherlands,^36^ Taiwan,^35^ and Australia.^78^ Thirty-nine reviews reported vision-related quality-of-life measures^13,15,16,19,21,22,23,24,25,26,27,28,29,30,31,32,33,34,37,42,44,54,55,58,59,60,61,62,66,67,68,69,70,71,73,74,75,76,77^; 7, disease-specific measures^35,38,39,40,41,57,65^; 4, generic measures^14,45,56,72^; and 10, more than 1 measure type.^17,18,20,36,43,46,63,64,78,79^ Reviews that assessed and reported the quality of the evidence either used the Grading of Recommendations, Assessment, Development, and Evaluations (GRADE) tool with 4 possible levels of evidence (very low, low, moderate, or high)^80^ or the US Preventive Services Task Force scale for overall quality of the evidence (poor, fair, or good).^81^

Findings about quality of life after ophthalmic interventions compared with quality of life at baseline or in a group receiving no intervention are presented in Table 2. Seven systematic reviews^13,14,15,16,17,18,19^ addressed interventions for age-associated cataract. One review^13^ found improvement in vision-related quality of life after treatment of early vision impairment attributable to cataract in 3 of the 5 studies included (with fair-quality evidence). Two reviews^14,15^ using results from the same primary studies showed improved vision-related quality of life in the group receiving expedited cataract surgery compared with the control group with a routine waiting time for surgery, and a third^16^ found benefits to immediate cataract surgery among people with AMD specifically, compared with those having no or delayed surgery (low-quality evidence). Two reviews^17,18^ with some overlap of primary studies found a nonclinically meaningful improvement in vision-related quality of life after a second eye cataract surgery compared with surgery in 1 eye only (with moderate-quality evidence in 1 review^17^). Both extracapsular cataract extraction with posterior chamber intraocular lens (IOL) implantation and intracapsular cataract extraction with aphakic glasses were associated with improvement in vision-related quality-of-life outcomes, but extracapsular cataract extraction resulted in greater improvement.^19^ Only 1 systematic review assessed interventions associated with refractive error. Using corrective lenses for vision impairment because of uncorrected refractive error improved vision-related quality of life (fair-quality evidence).^13^

Seven systematic reviews^13,20,21,22,23,24,54^ assessed interventions for AMD. Anti–vascular endothelial growth factor (anti-VEGF) therapy was associated with improved vision-related quality of life compared with no anti-VEGF therapy in 2 reviews,^13,14,15,16,17,18,19,20^ with some overlap in primary studies (described as mild to moderate improvements, with fair-quality evidence^13^ and moderate-quality evidence^20^). Treatment using aflibercept and ranibizumab resulted in improved vision-related quality of life from baseline to a similar extent (high-quality evidence that quality of life is similar in both groups).^21^ Macular surgery compared with observation (relative risk, 1.35 [95% CI, 1.09-1.68]; low-quality evidence),^22^ and antioxidant vitamin supplementation compared with placebo (low-quality evidence)^23^ were associated with improved vision-related quality of life, while radiotherapy compared with observation^54^ was not. Two reviews^23,24^ with some overlap of primary studies found that supplementation with lutein and/or zeaxanthin compared with placebo was not associated with better vision-related quality of life (with low-quality evidence in 1 review^23^).

Nine systematic reviews^25,26,27,28,29,30,31,32,33^ examined interventions for other retinal diseases. One systematic review reported that both anti-VEGF therapy and laser photocoagulation improved vision-related quality of life for diabetic retinopathy, but anti-VEGF therapy resulted in a greater improvement (moderate-quality evidence).^25^ Likewise, 3 reviews^26,27,28^ using identical primary studies found that anti-VEGF therapy, compared with a sham treatment, was associated with improvement in vision-related quality of life in people with macular edema secondary to central retinal vein occlusion (high-quality evidence in 1 review^27^ and moderate-quality evidence in another review^26^). Anti-VEGF therapy was also found to improve vision-related quality of life in those with branch retinal vein occlusion^29^ and choroidal neovascularization secondary to pathological myopia (moderate-quality evidence).^30^ Ocriplasmin injection compared with a sham treatment was associated with clinically meaningful improvement in vision-related quality of life among those with symptomatic vitreomacular traction^31^ and adhesion (moderate-quality evidence).^32^ In people with hereditary retinal dystrophies, vision-related quality of life was similar among those who received nutritional supplementation and placebo.^33^

Two systematic reviews^34,35^ addressed glaucoma interventions. Treatment of early open-angle glaucoma with laser trabeculoplasty and topical β-blockers compared with placebo was not associated with differences in vision-related quality of life.^34^ Selective laser trabeculoplasty and/or medication use did not result in changes in disease-specific quality of life in open-angle glaucoma.^35^

One systematic review^36^ addressed vision rehabilitation interventions. Compared with a passive control arm (delayed or no care), methods for enhancing vision (eg, low-vision service, customized prism glasses) resulted in an imprecisely estimated benefit in vision-related quality of life (very low-quality evidence), and multidisciplinary rehabilitation resulted in beneficial vision-related quality-of-life effects (with very low-quality evidence).^36^

One systematic review^37^ addressed vision screening. After-school vision screenings and the use of ready-made or custom-made spectacles both resulted in improvement in vision-related quality of life to a similar extent (with moderate-quality evidence that quality of life was similar in both groups).^37^

Subcutaneous immunotherapy for rhinoconjunctivitis was shown to result in greater improvement in rhinoconjuctivitis-specific quality of life compared with placebo among children in 1 systematic review^39^ (low-quality evidence) and among adults in another review^38^ (high-quality evidence). Sublingual immunotherapy was associated with better quality of life in 1 review^40^ (moderate-quality evidence), but not in another 1 that focused on children only.^39^ Intranasal fluticasone furoate was associated with better rhinoconjunctivitis-specific quality of life compared with placebo in people with allergic rhinoconjunctivitis.^41^

Two systematic reviews with some overlap of primary studies reported that adults with active uveitis who received adalimumab, relative to those receiving placebo, had a greater improvement in quality of life, but those with inactive uveitis did not have improvements in quality of life with therapy.^42,43^ Surgery for people with trichiasis improved vision-related quality of life regardless of perioperative azithromycin administration in 1 review.^44^ There were no differences in quality of life among people with thyroid eye disease who received radiotherapy or sham, according to 2 reviews^45,46^ based on results from the same primary study.

Thirty-three interventions among specific populations (eg, people with AMD, people with inactive disease, children only) were identified, after accounting for duplicate interventions assessed by multiple reviews and combining quality-of-life outcomes (health-related, vision-related, or disease-specific quality of life). A summary of findings is presented in Table 4. Overall, only 11 interventions^23,24,33,34,35,39,42,43,45,46,54^ were not associated with improved quality of life compared with baseline or compared with a group receiving no intervention: radiotherapy^54^ and supplementation with lutein and zeaxanthin^23,24^ for AMD, supplementation with nutrients for hereditary retinal dystrophies,^33^ early open-angle glaucoma treatment with laser trabeculoplasty and topical β-blockers,^34^ open-angle glaucoma treatment with selective laser trabeculoplasty and/or medications,^35^ adalimumab for the treatment of inactive uveitis (treatment improved quality of life among those with active disease),^42,43^ radiotherapy for the treatment of thyroid eye disease,^45,46^ and sublingual immunotherapy for rhinoconjunctivitis among children^39^ (however, this treatment improved quality of life in a review covering both children and adults^40^).

**Table 4.** Summary of Findings From Systematic Reviews of Ophthalmic Interventions

Area	Findings on quality of life	
	Improvement^a^	No difference
Age-related cataract	Cataract surgery for the treatment of early impairment in visual acuity (1); expedited cataract surgery vs awaiting cataract surgery (routine wait time) (2); expedited surgery vs routine wait time among people with age-related macular degeneration (1); second-eye surgery vs surgery in 1 eye only (2); cataract surgery by extracapsular cataract extraction with posterior chamber intraocular lens or intracapsular cataract extraction with aphakic glasses (1)	None
Cornea and refractive error	Corrective lenses for refractive error (1)	None
Age-related macular degeneration	Anti-VEGF vs no anti-VEGF (2); aflibercept or ranibizumab (1); macular surgery vs observation (1); multivitamin supplements vs placebo (1)	Radiotherapy vs observation (1); Lutein and/or zeaxanthin vs placebo (2)
Retina (other)	Anti-VEGF or laser photocoagulation for diabetic retinopathy (1); anti-VEGF vs sham for macular edema secondary to central retinal vein occlusion (3); anti-VEGF vs sham for macular edema secondary to branch retinal vein occlusion (1); anti-VEGF vs sham for choroidal neovascularization secondary to pathological myopia (1); ocriplasmin injection vs sham for symptomatic vitreomacular traction (1); ocriplasmin injection vs sham for symptomatic vitreomacular adhesion (1)	Nutritional supplementation vs placebo for hereditary retinal dystrophies (1)
Glaucoma	None	Laser trabeculoplasty and topic β-blockers vs placebo for early open glaucoma (1); Selective laser trabeculoplasty and medication or medication only for open-angle glaucoma (1)
Low vision	Multidisciplinary rehabilitation vs waiting list or no care (1); methods of enhancing vision (eg, low-vision service, customized prism glasses) vs waiting list or no care (1)	None
Vision screening	Ready-made spectacles or custom-made spectacles after school vision screening (1)	None
Rhinoconjunctivitis	Subcutaneous immunotherapy vs placebo (2); Sublingual immunotherapy vs placebo (all ages) (1); intranasal fluticasone furoate vs placebo for seasonal allergic rhinitis (1); intranasal fluticasone furoate vs placebo for perennial allergic rhinitis (1)	Sublingual immunotherapy vs placebo among children only (1)
Uveitis	Adalimumab vs placebo for active uveitis (2)	Adalimumab vs placebo for inactive uveitis (2)
Trichiasis	Surgery with or without preoperative azithromycin (1)	None
Thyroid eye disease	None	Radiotherapy vs sham (2)

Abbreviations: anti-VEGF, anti–vascular endothelial growth factor; QOL, quality of life.

^a^ Number of systematic reviews informing the intervention and outcome are shown in parentheses.

Comparisons of quality of life between 2 different ophthalmic interventions are presented in eTable 3 in the Supplement. Similar quality of life was reported across the interventions for most interventions compared. Interventions that were associated with small or moderate improvements in quality of life when compared with other interventions were immediate sequential cataract surgery compared with different date bilateral cataract surgery (moderate-quality evidence),^75^ multifocal IOLs compared with monofocal IOLs (very low-quality evidence in 1 review),^55,60,61^ toric IOLs compared with nontoric IOLs,^61^ macular translocation compared with photodynamic therapy (described as “insufficient evidence”^70^^(p2)^), the use of handheld electronic devices with optical devices compared with optical devices alone (moderate-quality evidence),^71^ vision rehabilitation using methods for enhancing vision (eg, low-vision service, customized prism glasses) compared with other interventions (moderate-quality evidence),^36^ and posterior lamellar tarsal rotation surgery for minor trichiasis compared with epilation.^44^

## Discussion

In this umbrella review, we performed a global, broad assessment of eye disease, vision impairment, and ophthalmic interventions on quality of life. There was a consistent association between vision impairment and eye disease with reduced quality of life across eye conditions, especially among adults. Seventy-five percent of ophthalmic interventions evaluated had evidence of a positive outcome on quality of life. Most notably, cataract surgery and the use of anti-VEGF therapy for AMD, diabetic macular edema, and macular edema secondary to other causes resulted in improved quality of life.

Vision impairment and eye diseases, namely glaucoma, diabetic retinopathy, AMD, and retinitis pigmentosa were associated with lower quality of life, using a range of outcome measures. Quantitative studies showed significant associations and sometimes a graded response, with worse vision impairment being associated with worse quality of life. Qualitative studies provided insight into the mechanisms of the associations, specifically on well-being and functioning. While the significant associations were expected, previous literature suggests that even more eye diseases are associated with worse quality of life. Notably, dry eye has been studied extensively, and a systematic review^82^ (excluded because of a lack of appropriate criteria for critical appraisal) has suggested that dry eye syndrome has a substantial association with reduced quality of life across countries in Europe, North America, and Asia.

Ophthalmic interventions differed in their association with quality of life. In general, treating cataract immediately after diagnosis, even in those with competing eye conditions such as AMD and who had already received a first cataract surgery, improved quality of life. Moreover, anti-VEGF therapy for a number of conditions, including AMD and diabetic macular edema, and the use of corrective lenses for refractive error were associated with improved quality of life. Projections from 2015 suggested that in 2020, 127.7 million people will have moderate or severe vision impairment because of uncorrected refractive error, 57.1 million because of cataract, 8.8 million because of AMD, and 3.2 million because of diabetic retinopathy.^83^ Three interventions (cataract surgery, corrective lenses, and anti-VEGF therapy) provide opportunities to improve the quality of life of more than 150 million individuals globally. Other interventions, such as treating rhinoconjunctivitis in children and adults, trichiasis in endemic areas, and uveitis in those with active and inactive disease, and low-vision rehabilitation, also have the potential to improve quality of life.

The 8 interventions that were not found to improve quality of life included 2 that focused on specific populations. This included the use of adalimumab, which did not improve quality of life among people with inactive uveitis but improved it in those with active disease, and sublingual immunotherapy for rhinoconjunctivitis, which did not improve quality of life among children but improved it when people of all ages were included in another review. Two of the interventions involved nutritional supplements; one was lutein or zeaxanthin for AMD, and the other was nutritional supplements for hereditary retinal dystrophies.

There were gaps in the evidence available on the outcomes of leading causes of visual impairment (eg, cataract, refractive error), among particular groups (eg, children, people from racial/ethnic minorities), and in low- and middle-income countries compared with high-income countries. Interventions for dry eye, refractive error, glaucoma, and diabetic retinopathy were underrepresented in this review in comparison with their prevalence globally. While systematic reviews about some topics may be lacking, there may be a lack of primary studies as well: 70% of the interventional systematic reviews that aimed to assess quality of life outcomes were subsequently excluded because they did not identify any primary studies reporting quality-of-life outcomes. Moreover, almost half of the interventions and outcomes identified in this review were comparing one intervention with another without presenting information about the change in quality of life from baseline in any of the groups, making it impossible to know whether any of the interventions had an association with quality of life to begin with. While this may be standard practice, as more and more eye diseases have well-established treatments, delaying treatment or using placebo control arms will not be possible. Thinking of ways to answer questions about potential advantages of interventions without depriving a group of beneficial therapy will be important. Researchers could be encouraged to analyze and present data such as overall changes from baseline values by treatment arm, or regardless of treatment arm, even if they are not the primary outcomes.

The review process highlighted the need for a unified definition for quality of life to study and understand the association with vision impairment and ophthalmic interventions on well-being and vision function from the patient perspective. Many systematic reviews were excluded because they considered patient satisfaction or patient-reported symptoms and discomfort as quality-of-life measures. While these measures fall under the umbrella of patient-reported outcomes and capture valuable information, they do not describe general well-being or vision function in day-to-day life. Moreover, in the included reviews, a wide range of quality of life measures were used between and within the systematic reviews, which limits the ability to compare findings between studies or combine them into meta-analyses.

### Limitations

Umbrella reviews findings are limited to results that have been synthesized in published reviews, which may be affected by publication bias themselves. Although this may have limited the availability of studies about specific topics, it is this approach that allowed for a global assessment of a broad topic in a systematic manner. Moreover, umbrella reviews are limited by the quality of the reviews and the data reported by the reviews; not all reviews reported specific estimates, tools used to measure outcomes, or enough context to interpret the results. However, we applied strict criteria for systematic reviews, including the working definition and critical appraisal criteria used, and excluded reviews with case series. This approach may have further limited the number of systematic reviews included, but it ensured the inclusion of reviews of higher quality. Finally, there were overlaps in primary studies used by the reviews, and many reviews reported findings based on results from 1 or 2 primary studies only; these were presented in both the text and Tables to allow readers to take them into account when interpreting the findings.

There is evidence that vision impairment and eye diseases in general are associated with lower quality of life, and most ophthalmic interventions are associated with improved quality of life. Reviews and primary studies addressing underrepresented diseases and reviews focusing on specific populations, such as people from low- and middle-income countries, are needed to expand generalizable knowledge on the association between eye health and quality of life.

## Conclusions

Vision impairment and eye conditions are associated with lower quality of life, and ophthalmic interventions can lead to significant improvement in quality of life. Scaling up interventions, such as cataract surgery, refractive error correction, and anti-VEGF therapy at a global level, has the potential to improve the quality of life of millions of people worldwide.

## Notes

Supplement. **eAppendix 1.** Preferred Reporting Items for Systematic Review and Meta-Analysis (PRISMA) reporting guidelines **eAppendix 2.** Changes to the Protocol **eAppendix 3.** MEDLINE Ovid Search Strategy **eFigure.** PRISMA Flow Chart **eTable 1.** Quality Appraisal Using the Joanna Briggs Institute Critical Appraisal Checklist for Systematic Reviews and Research Syntheses **eTable 2.** Systematic Review Characteristics (Selected from the Joanna Briggs Institute Data Extraction Form for Systematic Reviews and Research Syntheses) **eTable 3.** Comparisons of Quality of Life Impact of Different Ophthalmic Interventions by Systematic Review **eTable 4.** Studies Included eReferences.
