# Supplementary material for: A Global Assessment of Eye Health and Quality of Life: A Systematic Review of Systematic Reviews
Source: JAMA Ophthalmol. 2021 Feb 12;139(5):1–16. doi: 10.1001/jamaophthalmol.2021.0146 (PMC7881366; doi:10.1001/jamaophthalmol.2021.0146)
Supplement: Supplement. — eAppendix 1. Preferred Reporting Items for Systematic Review and Meta-Analysis (PRISMA) reporting guidelines eAppendix 2. Changes to the Protocol eAppendix 3. MEDLINE Ovid Search Strategy eFigure. PRISMA Flow Chart eTable 1. Quality Appraisal Using the Joanna Briggs Institute Critical Appraisal Checklist for Systematic Reviews and Research Syntheses eTable 2. Systematic Review Characteristics (Selected from the Joanna Briggs Institute Data Extraction Form for Systematic Reviews and Research Syntheses) eTable 3. Comparisons of Quality of Life Impact of Different Ophthalmic Interventions by Systematic Review eTable 4. Studies Included eReferences. [file jamaophthalmol-e210146-s001.pdf]

Supplemental Online Content

Assi L, Chamseddine F, Ibrahim P, et al. A global assessment of eye health and quality of life: a systematic review of systematic reviews. *JAMA Ophthalmol*. Published online February 12, 2021. doi:10.1001/jamaophthalmol.2021.0146

eAppendix 1. Preferred Reporting Items for Systematic Review and Meta-Analysis (PRISMA) reporting guidelines
eAppendix 2. Changes to the Protocol
eAppendix 3. MEDLINE Ovid Search Strategy
eFigure. PRISMA Flow Chart
eTable 1. Quality Appraisal Using the Joanna Briggs Institute Critical Appraisal Checklist for Systematic Reviews and Research Syntheses
eTable 2. Systematic Review Characteristics (Selected from the Joanna Briggs Institute Data Extraction Form for Systematic Reviews and Research Syntheses)
eTable 3. Comparisons of Quality of Life Impact of Different Ophthalmic Interventions by Systematic Review
eTable 4. List of Overlapping Studies Included in Table 2
eReferences.

This supplemental material has been provided by the authors to give readers additional information about their work.

© 2021 Assi L et al. *JAMA Ophthalmology.*

eAppendix 1. Preferred Reporting Items for Systematic Review and Meta-Analysis (PRISMA) reporting guidelines

Section/topic	#	Checklist item	Reported on page #
**TITLE**			
Title	1	Identify the report as a systematic review, meta-analysis, or both.	1
**ABSTRACT**			
Structured summary	2	Provide a structured summary including, as applicable: background; objectives; data sources; study eligibility criteria, participants, and interventions; study appraisal and synthesis methods; results; limitations; conclusions and implications of key findings; systematic review registration number.	3
**INTRODUCTION**			
Rationale	3	Describe the rationale for the review in the context of what is already known.	4
Objectives	4	Provide an explicit statement of questions being addressed with reference to participants, interventions, comparisons, outcomes, and study design (PICOS).	4
**METHODS**			
Protocol and registration	5	Indicate if a review protocol exists, if and where it can be accessed (e.g., Web address), and, if available, provide registration information including registration number.	4
Eligibility criteria	6	Specify study characteristics (e.g., PICOS, length of follow-up) and report characteristics (e.g., years considered, language, publication status) used as criteria for eligibility, giving rationale.	4, Table 1
Information sources	7	Describe all information sources (e.g., databases with dates of coverage, contact with study authors to identify additional studies) in the search and date last searched.	4
Search	8	Present full electronic search strategy for at least one database, including any limits used, such that it could be repeated.	4, eAppendix 3
Study selection	9	State the process for selecting studies (i.e., screening, eligibility, included in systematic review, and, if applicable, included in the meta-analysis).	4
Data collection process	10	Describe method of data extraction from reports (e.g., piloted forms, independently, in duplicate) and any processes for obtaining and confirming data from investigators.	5
Data items	11	List and define all variables for which data were sought (e.g., PICOS, funding sources) and any assumptions and simplifications made.	4-5
Risk of bias in individual studies	12	Describe methods used for assessing risk of bias of individual studies (including specification of whether this was done at the study or outcome level), and how this information is to be used in any data synthesis.	4-5

© 2021 Assi L et al. *JAMA Ophthalmology.*

Summary measures	13	State the principal summary measures (e.g., risk ratio, difference in means).	5
Synthesis of results	14	Describe the methods of handling data and combining results of studies, if done, including measures of consistency (e.g., I^2) for each meta-analysis.	5
Section/topic	#	Checklist item	Reported on page #
Risk of bias across studies	15	Specify any assessment of risk of bias that may affect the cumulative evidence (e.g., publication bias, selective reporting within studies).	NA
Additional analyses	16	Describe methods of additional analyses (e.g., sensitivity or subgroup analyses, meta-regression), if done, indicating which were pre-specified.	NA
**RESULTS**			
Study selection	17	Give numbers of studies screened, assessed for eligibility, and included in the review, with reasons for exclusions at each stage, ideally with a flow diagram.	5, eFigure
Study characteristics	18	For each study, present characteristics for which data were extracted (e.g., study size, PICOS, follow-up period) and provide the citations.	5-8, Tables 1, 2, eTable 2
Risk of bias within studies	19	Present data on risk of bias of each study and, if available, any outcome level assessment (see item 12).	eTable 1
Results of individual studies	20	For all outcomes considered (benefits or harms), present, for each study: (a) simple summary data for each intervention group (b) effect estimates and confidence intervals, ideally with a forest plot.	5-8, Tables 1, 2
Synthesis of results	21	Present results of each meta-analysis done, including confidence intervals and measures of consistency.	NA
Risk of bias across studies	22	Present results of any assessment of risk of bias across studies (see Item 15).	NA
Additional analysis	23	Give results of additional analyses, if done (e.g., sensitivity or subgroup analyses, meta-regression [see Item 16]).	NA
**DISCUSSION**			
Summary of evidence	24	Summarize the main findings including the strength of evidence for each main outcome; consider their relevance to key groups (e.g., healthcare providers, users, and policy makers).	9
Limitations	25	Discuss limitations at study and outcome level (e.g., risk of bias), and at review-level (e.g., incomplete retrieval of identified research, reporting bias).	10-11
Conclusions	26	Provide a general interpretation of the results in the context of other evidence, and implications for future research.	11
**FUNDING**			
Funding	27	Describe sources of funding for the systematic review and other support (e.g., supply of data); role of funders for the systematic review.	11

© 2021 Assi L et al. *JAMA Ophthalmology.*

eAppendix 2. Changes to the Protocol

1. The web-based software Covidence was used for the assessment of methodological quality and data collection instead of the JBI SUMARI software.
2. Findings about the different quality of life outcomes (health-related, vision-related, and condition-specific) were presented separately in the same descriptive tables, rather than in separate tables.
3. Summary of findings tables were not stratified according to the different quality of life outcomes (health-related, vision-related, or condition-specific).
4. The overall assessment of the evidence for each finding (such as GRADE) was presented in the text and descriptive tables but not in the summary of findings tables.

© 2021 Assi L et al. *JAMA Ophthalmology.*

eAppendix 3. MEDLINE Ovid Search Strategy

1. exp Eye Diseases/
2. exp Eye Injuries/
3. exp Administration, Ophthalmic/
4. exp Diagnostic Techniques, Ophthalmological/
5. exp Eye Protective Devices/
6. exp Glaucoma Drainage Implants/
7. exp Injections, Intraocular/
8. exp Ophthalmic Solutions/
9. exp Ophthalmologic Surgical Procedures/
10. exp Optical Devices/
11. exp Orbital Implants/
12. exp Orthoptics/
13. exp Pseudophakia/
14. exp Visual Prosthesis/
15. ((low* or handicap* or subnormal* or impair* or partial* or disab* or reduce* or diminish* or decrease*) adj3 (vision or visual* or sight*)).tw.
16. ((abnormal* or blurred or defect* or difficult* or dim or disturbed or hazy or interference or poor or tunnel or weak* or defect* or deficienc* or disorder* or disturb* or problem*) adj3 (vision or visual* or sight*)).tw.
17. ((delayed or agnosia or constriction* or prosthesis or prostheses) adj3 (vision or visual* or sight*)).tw.
18. ((vision or visual or sight*) adj2 loss).tw.
19. (Ocular or occular or intraocular or ophthalmol* or ophthalmic* or ophthalmop* or optic* or orbital or conjunctival or conjunctivitis or eye or eyes or eyelid* or cataract* or corneal or glaucoma* or lacrimal or lacrimation or macular or retinal or retinitis or retinoblastoma or retinopath* or retrobulbar neuritis or uveal or uveitis or vitrectomy or vitreous detachment* or vitreous haemorrhage* or vitreous hemorrhage* or vitreous membranes or vitreous strands or vitreous prolapse* or vitreous syneresis).tw.
20. (Amblyopia or Ametropia or Anisocoria or Anophthalmia or Anterior Chamber Haemorrhage or Anterior Chamber Hemorrhage or Aphakia or Aqueous Outflow Obstruction or Asthenopia or Balint's Syndrome or Blepharitis or Blepharospasm or chalazia or chalazion or Chorioretinal Disorder* or Chorioretinitis or Choroid Diseases or Choroidal or Choroiditis or Chromatopsia or Diplopia or Endophthalmitis or Epiphora or Episcleritis or Equatorial Staphyloma or Esotropia or Exophthalmos or Fixed Pupil* or Fuchs endothelial dystrophy or Hemianopia or Hemianopsia or Hepatolenticular Degeneration or Hordeola or Hordeolum or Horner's Syndrome or Hypopyon or Iritis or Keratitis or Keratoconjunctivitis or Keratoconus or Lens Disease* or Lens Disorder* or Lens Opacit* or Lens Subluxation or Localized Anterior Staphyloma or Meibomianitis or Miosis or Mydriasis or Myopia or Nystagmus or Oculopath* or Papilloedema or Periorbital Fat Herniation or (Periocular and carcinoma*) or Photalgia or Photophobia or Photopsia or Pigment Precipitation or Posterior capsule opacification or Posterior Dislocation Of Lens or Posterior Synechiae or Pseudophakia or Proliferative Vitreoretinopathy or Scleral Disease* or Scleral Staphyloma or Scleritis or Scotoma or Staphyloma Posticum or Strabismus or Symblepharon or Traumatic Hyphema or Wavefront Aberration* or Wegener's granulomatosis or Wilson's Disease or Xerophthalmia or refractive error* or near-sighted* or nearsighted* or short-sighted* or shortsighted* or hyperopia or farsighted* or far sighted* or long-sighted* or longsighted*or astigmatism or presbyopia* or onchocerciasis or onchocerciases).tw.
21. (LASIK or LASEK or Orthoptic* or "visual prosthesis" or "visual prostheses" or "artificial iris" or "capsular tension ring" or "cornea implant" or "intravitreal implant" or "lens implant" or "palpebral spring" or "punctal plug" or "retinal implant" or "sclerectomy implant" or glasses or spectacle* or "artificial lens" or "artificial implant lens" or pseudophakos or "orbit implant" or "ab interno gel implant" or "ab interno gel stent" or "anterior chamber drainage tube" or "aqueous drainage device" or "aqueous drainage implant" or "aqueous shunt" or glaukos or istent or keratoprosthesis).tw.
22. or/1-21
23. "Quality of Life"/ or Quality-Adjusted Life Years/ or "Value of Life"/ or Health Status/ or Sickness Impact Profile/ or Disability Evaluation/ or exp "Activities of Daily Living"/ or Cost-Benefit Analysis/ or "Surveys and Questionnaires"/ or Health surveys/ or exp psychometrics/
24. (quality adj2 life).tw.
25. ("disability adjusted life" or qaly* or qald* or qale* or qtime* or daly* or euroqol or "euro qol" or eq5d or "eq 5d" or hql or hqol or "h qol" or hrqol or "hr qol" or hye or hyes or health* year* equivalent* or hui or hui1 or hui2 or hui3 or "willingness to pay" or "standard gamble" or QOL or HRQL or HRQOL or wellbeing or "well being" or WHOQOL or "healthy days measures" or "EQ VAS" or "EQ 15D" or "36 Item Short Form Survey" or "SF 36" or "12 item Short Form Survey" or "SF 12" or "Visual Function Questionnaire" or "NEI VFQ" or "VFQ 25" or "IND VFQ 33" or "14 item Visual Functioning" or "VF 14" or "11 item Visual Functioning" or "VF 11" or "Impact of Vision Impairment" or IVI or "glaucoma utility index" or catquest or "Activities of Daily Vision Scale" or ADVS or "Cataract Symptom Scale" or "Daily Living Tasks Dependent Upon Vision" or DLTV or "Measure of Outcome in Ocular Disease" or "Refractive Status and Vision Profile" or "Vision Specific Sickness Impact Profile" or SIPV or "Visual Activities Questionnaire*" or VAQ or "Visual Disability Assessment*" or VDA or "Visual Disabilities Questionnaire*" or "Visual Function Questionnaire*" OR "VA LV VFQ" or "Glaucoma symptom scale" or "Symptom Impact Glaucoma Score" or GHPI or "Glaucoma Health Perceptions index").tw.
26. (health adj3 (utility* or disutili* or state or status)).tw.
27. ((visual or vision) adj3 (disabilit* or disabled or function* or activit* or task or performance or impairment or Questionnaire*)).tw.

© 2021 Assi L et al. *JAMA Ophthalmology.*

28. or/23-27
29. 22 and 28
30. Cochrane Database Syst Rev.ja. or Meta-Analysis.pt. or (Search* or Medline or (Systematic and Review)).tw.
31. limit 29 to systematic reviews
32. 30 or 31
33. exp Animals/ not exp Humans/
34. 32 not 33
35. 29 and 34

© 2021 Assi L et al. *JAMA Ophthalmology.*

eFigure. PRISMA Flow Chart

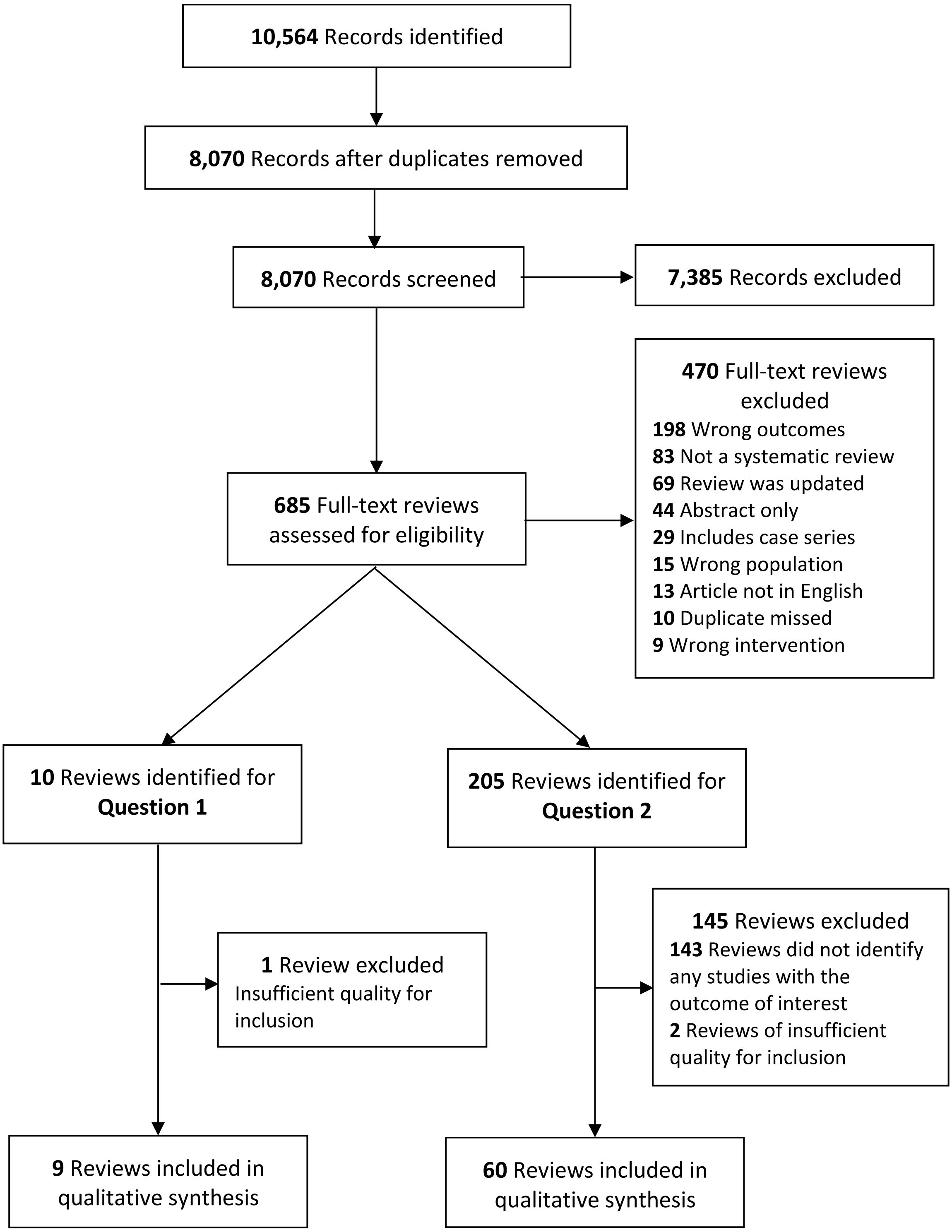

Moher D, Liberati A, Tetzlaff J, Altman DG, The PRISMA Group (2009). *P*referred *R*eporting *I*tems for *S*ystematic Reviews and *M*eta-*A*nalyses: The PRISMA Statement. PLoS Med 6(7): e1000097. doi:10.1371/journal.pmed1000097

© 2021 Assi L et al. *JAMA Ophthalmology.*

eTable 1. Quality Appraisal Using the Joanna Briggs Institute Critical Appraisal Checklist for Systematic Reviews and Research Syntheses

Systematic Review	1.Clear review question	2.Apppropriate inclusion criteria	3.Appropriate search strategy	4.Adequate sources searched	5.Appropriate criteria for appraising studies	6.Crtitical appraisal by 2 reviewers	7.Errors in data extraction minimized	8.Appropriate methods to combine studies	9.Assessment of publication bias	10.Practice recommen-dations supported by data	11.Appropriate research directives
Bennion 2012	Yes	Yes	Yes	Yes	Yes	Yes	Yes	Yes	NA (qualitative)	Yes	Yes
Brady 2016	Yes	Yes	Yes	Yes	Yes	Yes	Yes	Yes	NA (# of studies)	Yes	Yes
Braithwaite 2014	Yes	Yes	Yes	Yes	Yes	Yes	Yes	Yes	NA (# of studies)	Yes	Yes
Brito-Garcia 2017	Yes	Yes	Yes	Yes	Yes	Yes	Yes	Yes	NA (qualitative)	Yes	Yes
Burr 2012	Yes	Yes	Yes	Yes	Yes	Yes	Yes	Yes	NA (qualitative)	Yes	Yes
Burton 2015	Yes	Yes	Yes	Yes	Yes	Yes	Yes	Yes	No	Yes	Yes
Casparis 2017	Yes	Yes	Yes	Yes	Yes	Yes	Yes	Yes	NA (# of studies)	Yes	Yes
Chi 2020	Yes	Yes	Yes	Yes	Yes	Yes	Yes	Yes	Yes	Yes	Yes
Chou 2016	Yes	Yes	Yes	Yes	Yes	Yes	Yes	Yes	NA (# of studies)	Yes	Yes
Clarke 2018	Yes	Yes	Yes	Yes	Yes	Yes	Yes	Yes	No	Yes	Yes
Conner-Spady 2007	Yes	Yes	Yes	Yes	Yes	Yes	Yes	Yes	NA (qualitative)	Yes	Yes
D'Amanda 2020	Yes	Yes	Yes	Yes	Yes	Yes	unclear	Yes	NA (qualitative)	Yes	Yes
deSilva 2016	Yes	Yes	Yes	Yes	Yes	Yes	Yes	Yes	Yes	Yes	Yes
Eandi 2008	Yes	Yes	Yes	Yes	Yes	Yes	Yes	Yes	NA (# of studies)	Yes	Yes
Erekosima 2014	Yes	Yes	Yes	Yes	Yes	Yes	unclear	Yes	NA (missing data)	Yes	Yes
Evans 2010	Yes	Yes	Yes	Yes	Yes	Yes	Yes	Yes	NA (# of studies)	Yes	Yes
Evans 2017	Yes	Yes	Yes	Yes	Yes	Yes	Yes	Yes	NA (# of studies)	Yes	Yes
Evans 2018	Yes	Yes	Yes	Yes	Yes	unclear	Yes	Yes	No	Yes	Yes

© 2021 Assi L et al. *JAMA Ophthalmology.*

Systematic Review	1.Clear review question	2.Apppropriate inclusion criteria	3.Appropriate search strategy	4.Adequate sources searched	5.Appropriate criteria for appraising studies	6.Crtitical appraisal by 2 reviewers	7.Errors in data extraction minimized	8.Appropriate methods to combine studies	9.Assessment of publication bias	10.Practice recommen-dations supported by data	11.Appropriate research directives
Ford 2014	Yes	Yes	Yes	Yes	Yes	unclear	Yes	Yes	NA (qualitative)	Yes	Yes
Frampton 2014	Yes	Yes	Yes	Yes	Yes	Yes	Yes	Yes	NA (qualitative)	Yes	Yes
Garip 2019	Yes	Yes	Yes	Yes	Yes	Yes	Yes	Yes	NA (qualitative)	Yes	Yes
Giansanti 2009	Yes	Yes	Yes	Yes	Yes	Yes	Yes	Yes	NA (# of studies)	Yes	Yes
Herretes 2014	Yes	Yes	Yes	Yes	Yes	Yes	Yes	Yes	NA (qualitative)	Yes	Yes
Hodge 2007	Yes	Yes	Yes	Yes	Yes	No	Yes	Yes	NA (qualitative)	Yes	Yes
Ishikawa 2013	Yes	Yes	Yes	Yes	Yes	Yes	No	Yes	NA (qualitative)	Yes	Yes
Jin 2019	Yes	Yes	Yes	Yes	Yes	No	Yes	Yes	Yes	Yes	Yes
Kessel 2015	Yes	Yes	Yes	Yes	Yes	No	Yes	Yes	No	Yes	NA
Kessel 2016	Yes	Yes	Yes	Yes	Yes	Yes	No	Yes	No	Yes	NA
Khandelwal 2019	Yes	Yes	Yes	No	Yes	unclear	Yes	Yes	Yes	Yes	Yes
Khoo 2019	Yes	Yes	Yes	Yes	Yes	No	Yes	Yes	NA (qualitative)	Yes	Yes
Kim 2013	Yes	Yes	Yes	Yes	Yes	Yes	Yes	Yes	NA (qualitative)	Yes	Yes
Lake 2019	Yes	Yes	Yes	Yes	Yes	Yes	Yes	Yes	Yes	Yes	Yes
Lawrence 2015	Yes	Yes	Yes	Yes	Yes	Yes	Yes	Yes	NA (# of studies)	Yes	Yes
Lescrauwa et 2019	Yes	Yes	Yes	Yes	Yes	No	Yes	Yes	No	Yes	Yes
Li 2019	Yes	Yes	Yes	Yes	Yes	Yes	Yes	Yes	NA (# of studies)	Yes	Yes
Li 2020	Yes	Yes	Yes	Yes	Yes	No	Yes	Yes	NA (# of studies)	Yes	Yes
Lim 2016	Yes	Yes	Yes	Yes	Yes	Yes	Yes	Yes	Yes	Yes	Yes
Lin 2013	Yes	Yes	Yes	Yes	Yes	Yes	Yes	Yes	NA (missing data)	Yes	Yes
Liu 2014	Yes	Yes	Yes	Yes	Yes	Yes	Yes	Yes	Yes	Yes	Yes

© 2021 Assi L et al. *JAMA Ophthalmology.*

Systematic Review	1.Clear review question	2.Apppropriate inclusion criteria	3.Appropriate search strategy	4.Adequate sources searched	5.Appropriate criteria for appraising studies	6.Crtitical appraisal by 2 reviewers	7.Errors in data extraction minimized	8.Appropriate methods to combine studies	9.Assessment of publication bias	10.Practice recommen-dations supported by data	11.Appropriate research directives
Low 2019	Yes	Yes	Yes	Yes	Yes	Yes	Yes	Yes	No	Yes	No
Mitry 2013	Yes	Yes	Yes	Yes	Yes	Yes	Yes	Yes	NA (# of studies)	Yes	Yes
Neffendorf 2017	Yes	Yes	Yes	Yes	Yes	Yes	Yes	Yes	NA (# of studies)	Yes	Yes
Nyman 2010	Yes	Yes	Yes	No	Yes	No	No	Yes	NA (qualitative)	NA	Yes
Nyman 2012	Yes	Yes	Yes	Yes	Yes	No	No	Yes	NA (qualitative)	NA	Yes
Ollendorf 2013	Yes	Yes	Yes	Yes	Yes	No	No	Yes	No	Yes	Yes
Rajendram 2012	Yes	Yes	Yes	Yes	Yes	Yes	Yes	Yes	NA (# of studies)	Yes	Yes
Rees 2010	Yes	Yes	Yes	Yes	Yes	No	Yes	No	NA (qualitative)	Yes	Yes
Riaz 2006	Yes	Yes	Yes	Yes	Yes	Yes	Yes	Yes	No	Yes	Yes
Rodrigo 2011	Yes	Yes	Yes	Yes	Yes	Yes	Yes	Yes	Yes	Yes	Yes
Rolim de Moura 2007	Yes	Yes	Yes	Yes	Yes	Yes	Yes	Yes	No	Yes	Yes
Sarwar 2016	Yes	Yes	Yes	Yes	Yes	Yes	Yes	Yes	NA (# of studies)	Yes	Yes
Schakel 2019	Yes	Yes	Yes	Yes	Yes	Yes	No	Yes	Yes	Yes	Yes
Schuster 2013	Yes	Yes	Yes	Yes	Yes	No	Yes	Yes	Yes	Yes	No
Solomon 2019	Yes	Yes	Yes	Yes	Yes	Yes	Yes	Yes	NA (# of studies)	Yes	Yes
Spiteri 2013	Yes	Yes	Yes	Yes	Yes	Yes	No	Yes	No	Yes	Yes
Squires 2017	Yes	Yes	Yes	Yes	Yes	No	Yes	Yes	NA (qualitative)	Yes	Yes
Tseng 2018	Yes	Yes	Yes	Yes	Yes	Yes	Yes	Yes	NA (qualitative)	Yes	Yes
Urruticoechea-Arana 2019	Yes	Yes	Yes	Yes	Yes	Yes	No	Yes	NA (qualitative)	Yes	Yes

© 2021 Assi L et al. *JAMA Ophthalmology.*

Systematic Review	1.Clear review question	2.Apppropriate inclusion criteria	3.Appropriate search strategy	4.Adequate sources searched	5.Appropriate criteria for appraising studies	6.Crtitical appraisal by 2 reviewers	7.Errors in data extraction minimized	8.Appropriate methods to combine studies	9.Assessment of publication bias	10.Practice recommen-dations supported by data	11.Appropriate research directives
Van Nispen 2020	Yes	Yes	Yes	Yes	Yes	Yes	Yes	Yes	NA (# of studies)	Yes	Yes
Viani 2012	Yes	Yes	Yes	Yes	Yes	Yes	Yes	Yes	Yes	Yes	Yes
Virgili 2007	Yes	Yes	Yes	Yes	Yes	Yes	Yes	Yes	NA (# of studies)	Yes	Yes
Virgili 2018 a	Yes	Yes	Yes	Yes	Yes	Yes	Yes	Yes	Yes	Yes	Yes
Virgili 2018 b	Yes	Yes	Yes	Yes	Yes	Yes	Yes	Yes	No	Yes	Yes
Wang 2017 a	Yes	Yes	Yes	Yes	Yes	No	No	Yes	NA (# of studies)	Yes	No
Wang 2017 b	Yes	Yes	Yes	No	Yes	No	No	unclear	NA (qualitative)	NA	Yes
Xu 2017	Yes	Yes	Yes	Yes	Yes	No	No	Yes	No	Yes	No
Yang 2018	Yes	Yes	Yes	No	Yes	No	Yes	Yes	Yes	NA	Yes
Zhou 2014	Yes	Yes	Yes	Yes	Yes	No	Yes	Yes	Yes	Yes	No
Zhu 2016	Yes	Yes	Yes	Yes	Yes	Yes	Yes	Yes	NA (# of studies)	Yes	Yes

Abbreviations: NA, not applicable.

(1) Is the review question clearly and explicitly stated? (2) Were the inclusion criteria appropriate for the review question? (3) Was the search strategy appropriate? (4) Were the sources and resources used to search for studies adequate? (5) Were the criteria for appraising studies appropriate? (6) Was critical appraisal conducted by two or more reviewers independently? (7) Were there methods to minimize errors in data extraction? (8) Were the methods used to combine studies appropriate? (9) Was the likelihood of publication bias assessed? (10) Were recommendations for policy and/or practice supported by the reported data? (11) Were the specific directives for new research appropriate?

eTable 2. Systematic Review Characteristics (Selected from the Joanna Briggs Institute Data Extraction Form for Systematic Reviews and Research Syntheses)

Systematic Review	Primary Objective	Population	Intervention / Comparison	Outcomes	Date of search	Databases searched
Bennion 2012	To explore people's experiences of living with AMD and to ensure recommendations for practice fit with patients' demands	Elderly patients with macular degeneration	NA	"Experiences of AMD" assessed using qualitative methods	Apr 2012	Web of knowledge, PubMed, Science Direct, Psycarticles
D'Amanda 2020	To provide a systematic review on the psychosocial impacts (i.e., depression, anxiety, loneliness, psychological stress, and well-being) of Mendelian eye conditions to propose an overall model of illness factors, cultural factors, psychosocial impacts, and quality of life	Patients with a diagnosis of a Mendelian eye condition or their family members	NA	Psychological impacts including mental health (including depression, suicidality and anxiety), coping mechanisms, identity, and social impact including relationships and socioeconomic status	Mar 2018	CINHAL, Cochrane, Embase, PsychInfo, PubMed, Scopus, and Web of Science
Garip 2019	To identify coping strategies used by adults living with RP and to present how these findings may inform interventions to improve QOL in this population	Adults (aged 18 years and over) living with RP	NA	Participants' experiences of living or coping with RP, or RP impact on QOL	NR	Web of Science, PsycINFO, PsychArticles, Library Plus, Google Scholar, MEDLINE, CINAHL, PubMed, the Cochrane Library
Khoo 2019	To determine the relationship between diabetic retinopathy /diabetic macular edema and psychosocial functioning	Patients diagnosed with either type 1 or type 2 diabetes	NA	Prevalence, severity and level of psychosocial functioning, and incidence or progression of diabetic retinopathy /diabetic macular edema	Sep 2017	PubMed, Medline, Embase, Cochrane library

© 2021 Assi L et al. *JAMA Ophthalmology.*

Systematic Review	Primary Objective	Population	Intervention / Comparison	Outcomes	Date of search	Databases searched
Nyman 2010	To review the evidence for the presence of lower levels of psychosocial well-being in working-age adults with visual impairment and for interventions to improve such levels of psychosocial well-being	Working-age adults (mean age or at least 66% aged 18-59 years) who had visual impairment or were part of the supportive network (e.g., spouse of someone with visual impairment)	NA	Depression, mental health, anxiety, QOL, social functioning or social support	Jul 2008	PsycINFO, Medline
Nyman 2012	To synthesise the qualitative literature on how acquired visual impairment emotionally impacts older people in their daily lives and their views as to what factors inhibit/facilitate psychosocial adjustment to vision loss	Older people with irreversible vision loss	NA	Perceived psychosocial well-being or perceived inhibitors/facilitators to psychosocial adjustment to vision loss	Dec 2010	MEDLINE, EMBASE, PsycINFO, CINAHL
Schakel 2019	To compare fatigue levels between patients with visual impairment and controls with normal sight and to examine the association between fatigue and vision loss severity	Participants (aged ≥ 18 years) with at least moderate visual impairment according to the WHO criteria, defined as presenting VA worse than 20/60 and/or visual field worse than 30 degrees in the better-seeing eye, or on the basis of similar information or other indications of severe vision loss	NA	Fatigue severity or the prevalence of fatigue assessed by generic measures, fatigue outcomes compared to normally sighted controls and/or fatigue comparisons between patients with different levels of visual impairment according to VA	Apr 2019	PubMed, Embase.com, Ebsco/PsycINFO and Wiley/Cochrane Library

© 2021 Assi L et al. *JAMA Ophthalmology.*

Systematic Review	Primary Objective	Population	Intervention / Comparison	Outcomes	Date of search	Databases searched
Tseng 2018	To determine the relationship between hearing impairment, visual impairment, dual sensory impairment, and QOL	Older adults (aged over 65 years)	NA	QOL measures	Dec 2017	EMBASE, PubMed, CINAHL, MEDLINE, Cochrane Library, and Airiti Library
Wang 2017 a	To determine the impact of mild, moderate, and severe visual field loss on QOL in patients with glaucoma	Patients with glaucoma and a control group of participants without glaucoma	NA	Glaucoma visual field loss severity	Jun 2016	PubMed, EMBASE, CNKI PubMed, the Excerpta Medica database, the China National Knowledge Infrastructure, Google scholar
Brady 2016	To determine the efficacy and safety of steroid implants in people with chronic non-infectious posterior uveitis, intermediate uveitis, and panuveitis	Participants with better than hand-motion vision and history of chronic posterior uveitis, intermediate uveitis, or panuveitis (one eye with history of recurrent one year), both active and quiescent disease, and requiring systemic corticosteroids for more than one month or multiple sub-Tenon's capsule corticosteroid injections	Fluocinolone acetonide or dexamethasone intravitreous implants compared to standard-of-care therapy (e.g., systemic or intravitreal steroids, disease-modifying antirheumatic drugs)	**Primary**: proportion of participants with a recurrence of uveitis at 6 months; **secondary**: mean difference in BCVA, mean difference in QOL, and AEs	Nov 2015	CENTRAL, Ovid MEDLINE, Ovid MEDLINE In-Process and Other Non-Indexed Citations, Ovid MEDLINE Daily, Ovid OLDMEDLINE, EMBASE, PubMed, LILACS, mRCT, ClinicalTrials.gov, WHO ICTRP

© 2021 Assi L et al. *JAMA Ophthalmology.*

Systematic Review	Primary Objective	Population	Intervention / Comparison	Outcomes	Date of search	Databases searched
Braithwaite 2014	To investigate the effectiveness and safety of anti-VEGF therapies for the treatment of macular oedema secondary to central RVO	Participants of all ages who had unilateral or bilateral macular oedema secondary to central RVO	Anti-VEGF treatment compared to placebo (sham injection) or no treatment	**Primary**: proportion of participants with an improvement from baseline in BCVA of greater than or equal to 15 letters on the ETDRS Chart at 4 metres, after 6 months of follow-up, and any additional follow-up times; **secondary**: proportion of participants with a loss of 15 letters or more (ETDRS) compared to baseline, mean VA change, objective assessment of macular oedema regression measured by mean change in CRT on OCT, the number and type of complications relating to central RVO, the number of anti-VEGF or sham injections administered, the number and type of additional interventions administered, AEs, economic data, QOL (impact on health- or vision-related QOL or daily functioning) at 6 months and any additional follow-up times	Oct 2013	CENTRAL, Ovid MEDLINE, EMBASE, LILACS, CINAHL, OpenGrey, OpenSIGLE, mRCT, ClinicalTrials.gov, WHO ICTRP, Web of Science CPCIS
Brito-Garcia 2017	To review and assess the available scientific knowledge on the efficacy and safety of nutritional supplementation treatments in the group of HRDs	Children or adult patients diagnosed with hereditary retinal dystrophies	Nutritional supplements compared to standard of care, placebo, no treatment, or alternative treatment	Visual function (eg, VA, visual field and electroretinography parameters) and safety of interventions and/or patient-reported outcomes (eg, visual function, health-related QOL)	Nov 2016	Medline and PreMedline (OVIDinterface), EMBASE (Elsevier interface), SCI-EXPANDED (Web of Science interface), SSCI (Web of Scienceinterface), and the Cochrane Library limited to trials (Wiley interface)

© 2021 Assi L et al. *JAMA Ophthalmology.*

Systematic Review	Primary Objective	Population	Intervention / Comparison	Outcomes	Date of search	Databases searched
Burr 2012	To assess the effects of medication compared with initial surgery in adults with OAG	Patients aged 18 or abov with a diagnosis of OAG	Medical ocular hypotensive therapy compared to different surgical treatment modalities for OAG	**Primary**: progressive visual field loss, health-related quality of life; **secondary**: IOP reduction, progression of optic disc damage or nerve fibre layer loss, reduction of LogMAR score Snellen visual acuity, failure of randomised treatment, AEs, economic data	Aug 2012	CENTRAL, MEDLINE (Ovid MEDLINE,Ovid MEDLINE In-Process and Other Non-Indexed Citations, Ovid MEDLINE Daily, Ovid OLDMEDLINE), EMBASE, LILACS, BIOSIS, OpenGrey, CINAHL, Zetoc, mRCT, WHO ICTRP
Burton 2015	To assess the effects of interventions for trachomatous trichiasis for people living in endemic settings	Patients with trachomatous trichiasis	Any intervention intended to prevent corneal opacification from prolonged lash-globe contact compared to another intervention or to no treatment	**Primary**: post-operative trichiasis; **secondary**: VA change, corneal opacification change, acceptance of treatment, AEs, QOL measures, economic evaluation	May 2015	CENTRAL, Ovid MEDLINE, Ovid MEDLINE In-Process and Other Non-Indexed Citations, Ovid MEDLINE Daily, Ovid OLDMEDLINE, EMBASE, the ISRCTN registry, ClinicalTrials.gov, WHO ICTRP
Casparis 2017	To evaluate the effectiveness and safety of cataract surgery compared with no surgery in eyes with AMD	Participants whose eyes with AMD also had cataract that required cataract surgery	Cataract surgery compared to no or delayed surgery	**Primary**: BCVA in the operated eye and change in VA at one-year follow-up; **secondary**: progression of AMD in the operated eye, vision-related QOL measures, AEs	Dec 2016	CENTRAL, OvidMEDLINE, Epub Ahead of Print,In-Process & Other Non-Indexed Citations, Ovid MEDLINEDaily, Embase, LILACS, ISRCTN registry, ClinicalTrials.gov, WHO ICTRP

© 2021 Assi L et al. *JAMA Ophthalmology.*

Systematic Review	Primary Objective	Population	Intervention / Comparison	Outcomes	Date of search	Databases searched
Chi 2020	To compare SLT-related therapy with medication-only therapy in patients with OAG	Patients with OAG	SLT-related therapy (including both only SLT therapy and SLT + medication treatment) compared to medication-only treatments for OAG	IOP reduction, mean number of medications needed, success rate of IOP control, QOL parameters, AEs	Aug 2019	PubMed, Embase, Cochrane Library, Web of Science
Chou 2016	To update a 2009 systematic review on screening for impaired visual acuity among older adults for the USPSTF	Asymptomatic adults aged 65 years or older without known impaired visual acuity (based on current corrected vision) who have not sought care for evaluation of vision problems	Corrective lenses, reading aids, or photorefractive surgery due to uncorrected refractive errors; vitamin and oxidants and anti-VEGF for AMD; or cataract surgery	VA, vision-related QOL, functional capacity, mortality, cognition, harms	Feb 2015	Ovid MEDLINE, CENTRAL, Cochrane Database of Systematic Reviews
Clarke 2018	To assess the effects on vision of community vision screening of older people for visual impairment	People aged 65 years or above and are not identified as belonging to a particular risk group	Any attempt at population screening for visual impairment in a community setting, either vision alone or as part of a multi-component screening assessment	Degree of visual impairment in the population at the end of the trial	Nov 2017	CENTRAL, MEDLINE Ovid, Embase Ovid, ISRCTN registry, ClinicalTrials.gov, WHO ICTRP
Conner-Spady 2007	The study purpose was to synthesize the evidence regarding the relations among patient characteristics,WT, and health outcomes for patients on waiting lists for cataract surgery	Adults aged 18 or above with cataracts or were on the waiting list for or had a scheduled cataract surgery	Cataract surgery	VF-14, BCVA, AEs	2005	MEDLINE, EMBASE, Cochrane Library, EconLit, Social Sciences Abstracts

© 2021 Assi L et al. *JAMA Ophthalmology.*

Systematic Review	Primary Objective	Population	Intervention / Comparison	Outcomes	Date of search	Databases searched
deSilva 2016	To assess the visual effects of multifocal IOLs in comparison with the current standard treatment of monofocal lens implantation	Patients aged above 16 years undergoing cataract surgery and IOL implantation in one or both eyes	Any type of diffractive or refractive multifocal IOL compared to monofocal IOL implantation	**Primary**: distance/intermediate/near VA, unaided and corrected, spectacle dependence as reported by particpant; **secondary**: contrast sensitivity, PROMs including QOL or visual function measured by validated instruments, informal subjective assessment of visual function, patient satisfaction, glare, other optical aberrations, resource use and costs, AEs	Jun 2016	CENTRAL, Ovid MEDLINE, OvidMEDLINE In-Process and Other Non-Indexed Citations, Ovid MEDLINE Daily, Ovid OLDMEDLINE, Embase, the ISRCTN registry, ClinicalTrials.gov, WHO ICTRP
Eandi 2008	To examine the effects of macular translocation for CNV associated with AMD	People affected by CNV associated with AMD	Macular translocation compared to another treatment or no treatment	**Primary**: BCVA; **secondary**: contrast sensitivity, reading speed or any other validated measures of visual function, adverse outcomes, economic data, QOL	Jul 2008	CENTRAL, MEDLINE, EMBASE, LILACS
Erekosima 2014	To review the evidence for the effectiveness and safety of SCIT for treatment of adults with allergic rhinitis/rhinoconjunctivitis and/or asthma, focusing on SCIT formulations that are available in the U.S.	Patients with allergic rhinoconjunctivitis and/or allergic asthma due to aeroallergens confirmed by objective testing	SCIT alone or in combination with usual care (pharmacotherapy and environmental interventions) compared to placebo, other SCIT regimens, or pharmacotherapy	Symptom scores, medication scores, combined symptom and medication scores, QOL, safety (harm measures)	May 2012	MEDLINE, Embase, Cochrane Central Register of Controlled Trials, LILACS, Clinicaltrials.gov
Evans 2010	To examine the effects of radiotherapy on neovascular AMD	People with CNV secondary to AMD	Radiotherapy (no matter how it was delivered) compared to another treatment, low dosage irradiation, sham treatment or no treatment	**Primary**: loss of VA; **secondary**: measures of contrast sensitivity, new vessel growth, QOL measures, AEs	Mar 2010	CENTRAL, MEDLINE, EMBASE, LILACS, mRCT, ClinicalTrials.gov

© 2021 Assi L et al. *JAMA Ophthalmology.*

Systematic Review	Primary Objective	Population	Intervention / Comparison	Outcomes	Date of search	Databases searched
Evans 2017	To assess the effects of antioxidant vitamin or mineral supplementation on the progression of AMD in people with AMD	Patients with AMD in one or both eyes	Antioxidant vitamin or mineral supplementation, alone or in combination compared to placebo or no intervention	Progression to late AMD, neovascular AMD, geographic atrophy, progression to visual loss, QOL, resource use and cost, adverse events	Mar 2017	CENTRAL, MEDLINE Ovid, Embase Ovid, AMED, OpenGrey, ISRCTN registry, ClinicalTrials.gov, WHO ICTRP
Evans 2018	To evaluate the effectiveness of vision screening programmes carried out in schools to reduce the prevalence of correctable VA deficits due to refractive error in school-age children	School-age children and adolescents	VA assessment using any age-appropriate vision test	**Primary**: uncorrected, or suboptimally corrected, VA deficit due to refractive error 6 months after screening; **secondary**: VA deficit due to refractive error more than 6 months after screening, VA deficit due to causes other than refractive error, spectacle wearing, QOL	May 2017	CENTRAL, Ovid MEDLINE, Ovid Embase, ISRCTN registry, ClinicalTrials.gov, WHO ICTRP
Ford 2014	To review systematically the randomised controlled evidence for drug treatments of macular oedema secondary to central RVO	Patients with macular oedema secondary to central RVO	Pharmacological treatment compared to laser treatment, observation, placebo (sham injection) or another pharmacological intervention	**Primary**: mean change in BCVA or proportion of patients improving by 15 ETDRS letters or more; **secondary**: mean change in macular thickness using OCT, QOL measures, AEs	Mar 2013	MEDLINE, MEDLINE In-process, EMBASE, CDSR, DARE, HTA, NHSEED, CENTRAL
Frampton 2014	To assess the clinical effectiveness and cost-effectiveness of second-eye cataract surgery	Adults (aged ≥18 years) who had have one cataract operation already and still have or develop significant cataract-related visual impairment in the second eye	Cataract surgery for the second eye (any surgical technique) compared to cataract surgery in one eye only	Any measures of clinical vision (including measures of VA, contrast sensitivity and stereopsis), any PROMs of visual disability and symptoms, patient satisfaction with surgery and vision, health-related QOL, AEs	Jul 2013	Ovid MEDLINE, Ovid MEDLINE Daily Update, Ovid MEDLINE In-Process & Other Non-Indexed Citations, Ovid EMBASE, Web of Science: SCI-Expanded, CPCI-S, CPCI-SSH, BIOSIS Previews (Web of Science platform), CENTRAL, Cochrane Database of

© 2021 Assi L et al. *JAMA Ophthalmology.*

Systematic Review	Primary Objective	Population	Intervention / Comparison	Outcomes	Date of search	Databases searched
						Systematic Reviews, CRD, HTA
Giansanti 2009	To assess the effectiveness of submacular surgery for preserving or improving vision in patients with AMD	Patients affected by CNV associated with AMD	Submacular surgery compared to another treatment, sham treatment, or no treatment	**Primary**: BCVA after 1 year of follow up; **secondary**: contrast sensitivity, reading speed or any other validated measures of visual function, advserse outcomes, economic data, QOL	Feb 2009	CENTRAL, MEDLINE, EMBASE, LILACS
Herretes 2014	To assess the effectiveness and safety of corticosteroids as adjunctive therapy for bacterial keratitis, and to evaluate their effect on health economic outcomes and QOL outcomes	Patients diagnosed with bacterial keratitis clinically or microbiologically	Topical corticosteroids as an adjunct to antibiotics in the management of bacterial keratitis compared to placebo-controlled trials and trials that compared different steroids against each other as adjunctive agents	Primary outcomes: Clinical improvement, Clinical cure Secondary outcomes: microbiologic cure, time to clinical or microbiologic cure, adverse effects, QOL measures, economic data	Jul 2014	CENTRAL, Ovid MEDLINE, EMBASE, LILACS, mRCT, ClinicalTrials.gov, WHO ICTRP
Hodge 2007	To understand the relation between wait time for cataract surgery and patient outcomes and the variables that modify this relation	Studies performed in Canada or comparable regions (e.g., United Kingdom, Australia) to maximize the interpretability and generalizability of our review, studies using standardized and accepted assessment methods (e.g., slit lamp examination) and diagnostic criteria (e.g., Snellen or ETDRS acuity), studies on cataract removal among adults	Modern cataract surgery (wait time before cataract surgery)	Visual outcomes, AEs, QOL	Jun 2005	MEDLINE, HealthSTAR, EMBASE, CENTRAL, the Cochrane Database of Systematic Reviews, EconLit, NHS Economic Evaluation Database, HTA, Canadian Business and Current Affairs, Scopus, TRIP and the Cochrane Effective Practice, Organization of Care registry

© 2021 Assi L et al. *JAMA Ophthalmology.*

Systematic Review	Primary Objective	Population	Intervention / Comparison	Outcomes	Date of search	Databases searched
Ishikawa 2013	To appraise and synthesize evidence of the benefits of second-eye cataract extraction and to identify specific outcome variables that can be used to demonstrate need and effectiveness	Elderly population (aged 60 years or above) with first-eye and second-eye cataract surgeries	Second-eye cataract surgery	**Primary**: improvements in VA, contrast sensitivity, stereopsis, stereoacuity, field of vision, visual functioning; **secondary**: QOL, fall prevention, driving performance	Jan 2013	AgeLine, Academic Search Complete, CINAHL, PsycINFO, EconLit, MEDLINE via EBSCOhost, Ovid MEDLINE In-Process & Other Non-Indexed Citations, Ovid MEDLINE
Jin 2019	To compare the clinical performance of bifocal and trifocal IOLs in cataract surgery	Patients with age-related cataract who received cataract extraction with bifocal or trifocal intraocular lens implantation	Cataract extraction with bifocal intraocular lens implantation compared to cataract extraction with trifocal intraocular lens implantation	Visual performance evaluated as VA including uncorrected, corrected and distance-corrected performance, refraction cylinder, spherical equivalent refraction, spectacle independence, patient satisfaction after cataract surgery	Oct 2017	PubMed, Science direct, EMBASE
Kessel 2015	To examine the benefits and harms associated with immediate sequential bilateral cataract surgery with specific emphasis on the rate of complications, postoperative anisometropia, and subjective visual function in order to formulate evidence-based national Danish guidelines for cataract surgery	Patients with bilateral age-related cataract undergoing phacoemulsification	Immediate sequential bilateral cataract surgery compared to surgery on separate days	Number of AEs, serious AEs (specifically the number of sight-threatening complications), postoperative anisometropia (>2 diopters difference in spherical equivalent), patient's subjective satisfaction with the procedure	Sep 2014	Embase, PubMed, Cochrane Central

© 2021 Assi L et al. *JAMA Ophthalmology.*

Systematic Review	Primary Objective	Population	Intervention / Comparison	Outcomes	Date of search	Databases searched
Kessel 2016	The aim of this study was to provide evidence-based recommendation on which patients with age-related cataract are most likely to benefit from surgery	Patients with age-related cataract and poor preoperative VA (20/40 or lower) compared to patient with fair preoperative VA (better than 20/40) patient with age-related cataract and fair preoperative VA (≥20/40) compared with the patient with poor preoperative VA (<20/40) but few or no subjective cataract-related complaints	Cataract surgery	**Primary**: benefit, defined as an improvement in objective VA (2 Snellen lines or greater or a doubling of the visual angle or improvement as defined by the included studies) or subjective visual function assessed by validated questionnaires; **secondary**: harms of surgery, defined as peri- or postoperative complications as reported by included studies	Aug 2014	EMBASE, MEDLINE, CINAHL, Cochrane Library
Khandelwal 2019	To assess visual outcomes in patients receiving multifocal IOLs compared to either monofocal IOLs or monovision, and to compare results between newer and older IOLs	Adult patients undergoing cataract extraction	Multifocal lens compared to a standard monofocal lens or monovision	**Primary**: spectacle independence; **secondary**: corrected and uncorrected distance vision, uncorrected near vision, vision function, QOL, harms	Apr 2017	PubMed
Kim 2013	The objective of the current systematic review was to summarize the evidence regarding the efficacy and safety of SCIT and SLIT for the treatment of pediatric asthma and allergic rhinoconjunctivitis	Children with allergic asthma and/or rhinoconjunctivitis due to inhalant allergens, with diagnoses confirmed by using objective testing (positive result on skin allergy testing and/or in vitro specific immunoglobulin E allergy testing)	SCIT formulations available in the U.S. or SLIT formulations with close off-label substitutes, alone or in combination with usual care, and compared to placebo, pharmacotherapy, or other SIT regimens	Clinical outcomes or safety	May 2012	Medline, Embase, LILACS, CENTRAL

© 2021 Assi L et al. *JAMA Ophthalmology.*

Systematic Review	Primary Objective	Population	Intervention / Comparison	Outcomes	Date of search	Databases searched
Lake 2019	To assess the effects of toric IOLscompared with LRIs in the management of astigmatism during phacoemulsification cataract surgery	People with astigmatism who are having cataract surgery by phacoemulsification	Toric IOL compared to LRIs	**Primary**: proportion of participants with postoperative residual refractive astigmatism of less than 0.50 dioptres, mean postoperative residual refractive astigmatism in diotropes; **secondary**: mean postoperative uncorrected distance VA, spectacle independence for distance, vision-related QOL, and adverse effects	Sep 2019	CENTRAL, MEDLINE Ovid, Embase Ovid, ISRCTN registry, US National Institutes of Health Ongoing Trials Register Clinical Trials.gov, WHO ICTRP, Medline and Embase economic search
Lawrence 2015	To provide authoritative, reliable evidence regarding the safety, feasibility, effectiveness and cost-effectiveness of day case cataract extraction by comparing clinical outcomes, cost-effectiveness, patient satisfaction or a combination of these in cataract operations performed in day care versus in-patient units	People with age-related cataract	Cataract extraction and IOL implantation done as day cases compared to cataract extraction and IOL implantation done as in-patient cases	**Primary**: achievement of BCVA 6/18 or better in operated eye 6 weeks after surgery; **secondary**: adverse effects, intraoperative complications, postoperative complications, QOL measures, economic data	Aug 2015	CENTRAL, Ovid MEDLINE, Ovid MEDLINE In-Process and Other Non-Indexed Citations, Ovid MEDLINE Daily, Ovid OLDMEDLINE, EMBASE, LILACS, ISRCTN registry, ClinicalTrials.gov, WHO ICTRP
Lescrauwaet 2019	To summarize, and where possible synthesize, the patient reported outcomes relating to the use of ocriplasmin for the treatment of vitreomacular traction	Patients with a diagnosis of symptomatic vitreomacular adhesion, including vitreomacular traction and macular holes	Intravitreal injection of ocriplasmin compared to natural history, intravitreal placebo, sham or gas injection	PROMs	Oct 2018	PubMed MEDLINE, Elsevier Embase, CENTRAL

© 2021 Assi L et al. *JAMA Ophthalmology.*

Systematic Review	Primary Objective	Population	Intervention / Comparison	Outcomes	Date of search	Databases searched
Li 2019	To assess the effectiveness and safety of conventional occlusion versus atropine penalization for amblyopia	Participants of any age with either unilateral strabismic, anisometropic, or mixed (strabismicrefractive) amblyopia	Conventional occlusion versus atropine penalization for amblyopia	**Primary**: mean difference in VA of the amblyopic eye on at 12 months from commencement of treatment; **secondary**: change in binocular function, data on QOL outcomes, economic data, data on harm due to the intervention	Sep 2018	CENTRAL, MEDLINE, EMBASE, LILACS, WHO ICTRP
Li 2020	To investigate the effects of monthly versus non-monthly intravitreous injection of an anti-VEGF agent in people with newly diagnosed neovascular AMD	People with neovascular age-related macular degeneration	Monthly intravitreous injection of different anti-VEGF agents compared to non-monthly (PRN) intravitreous injection of different anti-VEGF agents	Change in BCVA at 1 year, gain of ≥ 15 letters visual acuity at 1 year, change in CRT at 1 year, change in QOL scores at 1 year, number of injections at 1 year, cost of treatment per person at 1 year, endophthalmitis	Oct 2019	CENTRAL, MEDLINE, Embase, LILACS, three trials registers
Lim 2016	To answer the question: is there evidence to support the prophylactic use of topical NSAIDs either in addition to, or instead of, topical steroids postoperatively to reduce the incidence of macular oedema and associated visual morbidity	Adult participants that had undergone standard surgery for age-related cataract	Preoperative and/or postoperative topical NSAIDs alone or in conjunction with postoperative topical steroids compared to postoperative topical steroids alone	The proportion of people with a poor vision outcome due to macular oedema in the study eye at 3 months after surgery, QOL or patient satisfaction measure, change in CRT from preoperative assessment in the study eye, at three months and 12 months after surgery, as measured by OCT scan	Sep 2016	CENTRAL, Ovid MEDLINE, Embase, LILACS, ISRCTN registry, ClinicalTrials.gov, WHO ICTRP
Lin 2013	To systematically review the effectiveness and safety of aqueous SLIT for allergic rhinoconjunctivitis and asthma	Patients with allergic rhinoconjunctivitis and/or allergic asthma due to airborne allergens	SLIT delivered as an aqueous solution compared to placebo, other SLIT regimens, or pharmacotherapy	**Primary**: symptom scores (for rhinitis, conjunctivitis, or asthma), medication scores, combined symptom and medication scores, QOL, safety or harms, and AEs; **secondary**: pulmonary function test results and provocational test results (allergen challenge)	Dec 2012	MEDLINE, EMBASE, LILACS, CENTRAL
Liu 2014	To evaluate the effects of lutein and zeaxanthin on visual function in RCTs of AMD patients	Patients diagnosed with AMD and randomized to receive lutein and/or zeaxanthin or placebo	Lutein and/or zeaxanthin compared to placebo	Visual function variables, including VA, contrast sensitivity, glare recovery time, score of Visual Function Questionnaire	Apr 2014	PubMed, EMBASE, Web of Science, Cochrane Library database

© 2021 Assi L et al. *JAMA Ophthalmology.*

Systematic Review	Primary Objective	Population	Intervention / Comparison	Outcomes	Date of search	Databases searched
Low 2019	To compare the effects of aflibercept, bevacizumab and ranibizumab on BCVA changes, QOL and ocular or systemic AEs in patients with neovascular AMD, diabetic macular oedema and central or branch RVO	Patients with neovascular AMD, diabetic macular oedema and central or branch RVO	Comparing at least two anti-VEGF agents (aflibercept, bevacizumab or ranibizumab)	VA, functional status, QOL measures, systemic AEs, ocular harms or cost-effectiveness, cost outcomes in the U.S.	Feb 2017	Ovid MEDLINE, PubMed, Elsevier EMBASE, Ovid EMB Reviews, trial registries, regulatory agency websites
Mitry 2013	To investigate the efficacy and gather evidence from RCTs on the safety of anti-VEGF agents for the treatment of macular oedema secondary to branch RVO	Participants of all ages and both genders who have had unilateral or bilateral macular oedema secondary to branch RVO	Anti-VEGF treatment compared with another treatment, no treatment, or placebo	**Primary**: improvement from baseline in BCVA of greater than or equal to 15 letters on the ETDRS Chart at six months and at 12 months of follow-up; **secondary**: mean VA change at six months and any additional follow-up intervals reported, the proportion of participants with a loss of 15 or more letters (ETDRS) compared with baseline, at six months and any additional follow-up intervals, change in CRT on OCT from baseline and final reported follow-up, the number and type of complications, the number of additional interventions administered, QOL outcomes, economic data, adverse outcomes	Aug 2012	CENTRAL, part of The Cochrane Library, Ovid MEDLINE, EMBASE, LILACS, mRCT, ClinicalTrials.gov, WHO ICTRP

© 2021 Assi L et al. *JAMA Ophthalmology.*

Systematic Review	Primary Objective	Population	Intervention / Comparison	Outcomes	Date of search	Databases searched
Neffendorf 2017	To assess the efficacy and safety of ocriplasmin compared to no treatment, sham or placebo for the treatment of symptomatic vitreomacular adhesion	People with symptomatic vitreomacular adhesion, including vitreomacular traction and macular holes of 400 µm or less with persisting vitreomacular adhesion	Intravitreal ocriplasmin 125 µg injection compared to placebo or sham injection (control)	Proportion of eyes with complete release of vitreous adhesion (by OCT), proportion of eyes with closure of macular hole, proportion of eyes with complete posterior vitreous detachment, proportion of eyes with 3 or more line improvement in BCVA, proportion of eyes requiring pars plana vitrectomy within six months of ocriplasmin	Feb 2017	CENTRAL, MEDLINE Ovid, Embase Ovid, PubMed, ISRCTN registry, US National Institutes of Health Ongoing Trials Register, WHO ICTRP
Ollendorf 2013	To evaluate the comparative effectiveness of anti-vascular endothelial growth factor therapy in the treatment of diabetic macular edema	Patients with any form of diabetic macular edema, including focal, diffuse, and clinically significant macular edema	Ranibizumab, bevacizumab, aflibercept, or pegaptanib, compared to the comparator of primary interest was focal or grid laser photocoagulation, the traditional gold standard therapy for patients with diabetic macular edema	VA, health-related QOL, generic and vision-specific assessments of health-related QOL	Jun 2012	MEDLINE, EMBASE, CENTRAL
Rajendram 2012	To review current evidence from RCTs for the effectiveness and short-term adverse events of orbital radiotherapy in adult TED when compared to sham radiotherapy, other interventions and glucocorticoids	Adults (aged 18 years old or above) with clinically diagnosed TED	Orbital radiotherapy of any dose and duration compared with sham radiotherapy or other intervention; orbital radiotherapy combined with glucocorticoids compared with glucocorticoids alone	Treatment successes, number of post-treatment rehab surgical procedures to correct functional disability, disease severity score, disease activity score, AEs, economic data, QOL measures	Mar 2012	CENTRAL, MEDLINE, EMBASE, LILACS, mRCT, ClinicalTrials.gov, WHO ICTRP
Rees 2010	To outline current evidence for the impact of low-vision rehabilitation programs on psychological well-being	Participants aged 18 years and above with VA of less than 6/12 or significant visual field loss	Multidisciplinary low-vision services, individual low-vision rehabilitation service components, and specifically developed group psychosocial programs or individual psychological interventions	Scales or subscales assessing mental health, psychological scales or subscales assessing mental health, psychological symptoms (e.g., anxiety and/or depression), or measures of vision-specific distress or adjustment	Feb 2010	OVID Medline, OVID CINAHL, CSA Illumina PsycINFO, CSA Illumina Social Services Abstracts

© 2021 Assi L et al. *JAMA Ophthalmology.*

Systematic Review	Primary Objective	Population	Intervention / Comparison	Outcomes	Date of search	Databases searched
Riaz 2006	To compare the effects of different surgical interventions for age-related cataract	People with age-related cataract	Phacoemulsification with a posterior chamber lens implant; manual small incision cataract surgery with a posterior chamber lens implant; extracapsular extraction with or without a posterior chamber intraocular lens implant; intracapsular extraction with or without an anterior chamber intraocular lens implant	**Primary**: late postoperative VA; **secondary**: early postoperative VA, complications during surgery, complications at one year or more after surgery, corneal endothelial cell loss, visual function other than VA, QOL, costs	Jul 2006	CENTRAL, MEDLINE, EMBASE up, NRR
Rodrigo 2011	To confirm and clarify the magnitude and clinical significance of the effect of intranasal fluticasone fuorate in patients with allergic rhinitis.	Children (aged ≤12 years) and adolescents-adults (aged >12 years) with seasonal or perennial allergic rhinitis (diagnosis confirmed by the clinical history or the allergen identified, and sensitivity proven by positive skin prick test)	Administration of topical fluticasone fuorate at any dose over any period of time compared with a placebo	**Primary**: reflective and instantaneous total ocular symptom scores, reflective and instantaneous total nasal symptom scores; **secondary**: assessment of response to therapy, QOL, AEs	Oct 2010	MEDLINE, EMBASE, CENTRAL
Rolim de Moura 2007	To investigate the effects of laser trabeculoplasty for treating OAG when compared to medication, incisional glaucoma surgery or no intervention	People with any diagnosis of OAG (primary, secondary pigment dispersion, corticosteroid-induced glaucoma and exfoliation or pseudoexfoliation syndromes)	Laser trabeculoplasty technique compared to one or more of the following: medical ocular hypotensive therapy, laser trabeculoplasty combined with medical ocular hypotensive therapy, glaucoma drainage surgery, an alternative laser trabeculoplasty technique, no intervention	**Primary**: failure to control IOP, failure to stabilize visual field progression, failure to stabilize optic neuropathy; **secondary**: necessity of adding or changing the medical therapeutic regimen, adverse effects, QOL measures, economic data	Jun 2007	CENTRAL, MEDLINE, EMBASE, LILACS

© 2021 Assi L et al. *JAMA Ophthalmology.*

Systematic Review	Primary Objective	Population	Intervention / Comparison	Outcomes	Date of search	Databases searched
Sarwar 2016	To assess and compare the effectiveness and safety of intravitreal injections of aflibercept versus ranibizumab, bevacizumab, or sham for treatment of patients with neovascular AMD	Patients with diagnosed subfoveal neovascular AMD, confirmed by fluorescein angiography, who received no previous treatment for AMD in the study eye	Aflibercept monotherapy compared to ranibizumab, bevacizumab, or sham treatment	**Primary**: mean change from baseline in number of letters of BCVA; **secondary**: mean change in number of letters of BCVA at two years, proportion of participants who gained/lost 15 or more letters of BCVA, proportion of participants with BCVA worse than 20/200, proportion of eyes with absence of fluid on OCT, proportion of eyes with absence of leakage on fluorescein angiography, mean number of injections received, mean change in CRT, mean change in extent of CNV, QOL, AEs	Nov 2015	CENTRAL, Ovid MEDLINE, EMBASE, PubMed, LILACS, mRCT, ClinicalTrials.gov, WHO ICTRP
Schuster 2013	To provide a summary of the impact on vision of an aspheric IOL compared with a spherical IOL in cataract surgery	Patients from published RCTs undergoing cataract surgery	Cataract surgery with aspheric compared with spherical monofocal IOL implantation	BCVA, contrast sensitivity, subjective perception of the quality of vision	May 2011	MEDLINE, EMBASE, Web of Science, BIOSIS, Cochrane Library databases

© 2021 Assi L et al. *JAMA Ophthalmology.*

Systematic Review	Primary Objective	Population	Intervention / Comparison	Outcomes	Date of search	Databases searched
Solomon 2019	To investigate ocular and systemic effects of, and quality of life associated with, intravitreous injection of three anti-VEGF agents (pegaptanib, ranibizumab, and bevacizumab) versus no anti-VEGF treatment for patients with neovascular AMD and to compare the relative effects of one of these anti-VEGF agents versus another when administered in comparable dosages and regimens	Patients with neovascular AMD	Intravitreal injections of anti-VEGF agents compared to another treatment, sham treatment, or no treatment	**Primary**: BCVA; **secondary**: VA outcomes, any other measures of visual function, assessment of morphologic characteristics, QOL measures, economic data, adverse outcomes	Jan 2018	CENTRAL, MEDLINE Ovid, Embase Ovid, LILACS, ISRCTN, ClinicalTrials.gov, WHO ICTRP
Spiteri 2013	To determine whether internal limiting membrane peeling improves anatomical and functional outcomes of macular hole surgery compared with the no-peeling technique and to investigate the impact of different parameters such as presenting vision, stage/size of the hole and duration of symptoms in the success of the surgery	Patients with idiopathic full-thickness macular hole at stages 2, 3 and 4	Macular hole surgery with internal limiting membrane peeling compared to macular hole surgery without internal limiting membrane peeling	**Primary**: best-corrected distance VA at 6 months postoperatively; **secondary**: best-corrected distance VA at 3 and 12 months, best-corrected near VA at 3, 6, and 12 months, primary (after a single surgery) and final (after >1 surgery) macular hole closure, need for additional surgical interventions, intraoperative and postoperative complications, PROMs, cost-effectiveness	Feb 2013	CENTRAL, Ovid MEDLINE, Ovid MEDLINE In-Process and Other Non-Indexed Citations, Ovid MEDLINE Daily, Ovid OLDMEDLINE, EMBASE, LILACS, mRCT, ClinicalTrials.gov, WHO ICTRP

© 2021 Assi L et al. *JAMA Ophthalmology.*

Systematic Review	Primary Objective	Population	Intervention / Comparison	Outcomes	Date of search	Databases searched
Squires 2017	To assess the clinical effectiveness and safety of adalimumab subcutaneous injection and dexamethasone intravitreal implant within their marketing authorisations in adults with non-infections intermediate uveitis, posterior uveitis or panuveitis	Adults (aged ≥ 18 years) with non-infectious intermediate uveitis, posterior uveitis or panuveitis	Adalimumab (subcutaneous injections), dexamethasone intravitreal implant	VA, improvement in disease activity, uveitis-related tissue damage or complications, reduction in systemic steroid use, mortality, adverse effects of treatment, health-related QOL, including generic measures and functional measures, composite end points incorporating more than one of the above	Jun 2016	MEDLINE, MEDLINE Epub Ahead of Print, MEDLINE In-Process & Other Non-Indexed Citations, EMBASE, Cochrane Database of Systematic Reviews, Database of Abstracts of Reviews of Effects, CENTRAL, HTA, NHS Economic Evaluation Database, CINAHL, CPCI, WHO ICTRP
Urruticoechea-Arana 2019	To compare the efficacy and safety of biological therapy with cyclosporin A, azathioprine, or placebo in uveitis flares and other ocular outcomes in patients with Behçet disease	Adult patients with Behçet's and uveitis on biological therapies compared to patients on placebo or active control with cyclosporin A or azathioprine	Treatment with biological therapies defined as those drugs that were developed to be directed highly specifically at particular well-defined molecules expressed on cells or secreted into the extracellular space; treatment with cyclosporin A, azathioprine, or placebo	Number of uveitis flares, macular edema, and safety outcomes	Aug 2017	MEDLINE (PubMed) , EMBASE, Cochrane Library (Wiley Online)

© 2021 Assi L et al. *JAMA Ophthalmology.*

Systematic Review	Primary Objective	Population	Intervention / Comparison	Outcomes	Date of search	Databases searched
Van Nispen 2020	To assess the effectiveness of low vision rehabilitation interventions on health-related QOL, vision-related QOL or visual functioning and other closely related PROMs in visually impaired adults	Adults (≥ 18 years) of either gender, with a vision impairment according to the WHO 2007 definition. Included studies had to be about vision impairment of irreversible nature, which was defined as a duration of at least 6 months	Rehabilitation interventions with waiting lists or no care, or, usual or other care	**Primary**: QOL using validated one-dimensional or multidimensional questionnaires; **secondary**: PROMs closely related to QOL concerning health and well-being, including physical and functional measures (e.g.activities of daily living, mobility and orientation, reading), psychological measures (e.g. depression, mood, anxiety, adaptation to vision loss, self-esteem), social measures (e.g.loneliness or independence), adverse outcomes	Sep 2019	CENTRAL, MEDLINE Ovid, Embase Ovid, CINAHL EBSCO, PsycINFO Ovid, ISRCTN registry, US National Institutes of Health Ongoing Trials Register ClinicalTrials.gov, WHO ICTRP
Viani 2012	To evaluate the efficacy of radiotherapy with total dose of 20 Gy in the treatment of Graves' ophthalmopathy	Patients with mild or moderate Graves Ophthalmopathy, diagnosed for the first time and not resistant to previous treatment	Primary treatment with radiotherapy with total dose of 20 Gy with or without a glucocorticoid of any type compared to primary treatment with radiotherapy with total dose different from 20 Gy, no radiotherapy or any another treatment	Efficacy of radiotherapy, response to radiotherapy (defined as clinical success according to each trial), QOL, AEs	Jul 2006	Medline, EMBASE, Cochrane library
Virgili 2007	To examine the effects of laser photocoagulation for neovascular AMD	Patients affected by CNV associated with AMD	Laser photocoagulation compared to no treatment or sham treatment, in addition to different photocoagulation techniques	**Primary**: VA, contrast sensitivity; **secondary**: reading ability measured with any reading chart, performance in real-world or laboratory vision-related tasks other than reading, QOL measures using QOL vision-specific questionnaires, AEs, economic data	Mar 2007	CENTRAL, MEDLINE, EMBASE, LILACS, NRR, ZETOC

© 2021 Assi L et al. *JAMA Ophthalmology.*

Systematic Review	Primary Objective	Population	Intervention / Comparison	Outcomes	Date of search	Databases searched
Virgili 2018 a	To compare the effectiveness and safety of the different anti-VEGF drugs in preserving and improving vision and quality of life in people with diabetic macular oedema using network meta-analysis methods	Patients with diabetic macular edema for whom anti-VEGF treatment is indicated	Any antiangiogenic drug with anti-VEGF modalities compared to another drug with anti-VEGF modalities, laser treatment, sham treatment or no treatment	**Primary**: BCVA expressed as the proportion of participants with at least 15 ETDRS letters of improvement in BCVA from baseline to 12 months; **secondary**: mean change in BCVA, mean change in CRT, mean change in QOL, AEs	Apr 2017	CENTRAL, MEDLINE Ovid, Embase Ovid, LILACS, ISRCTN registry, ClinicalTrials.gov, ICTRP
Virgili 2018 b	To assess the effects of different visual reading aids for adults with low vision	Patients aged 16 or above with low vision as defined by study investigators	Any device or aid used for reading visually (reading aids that maximise the person's visual reading capacity, including non-electronic aids, that is, optical devices such as magnifiers and telescopes, and electronic aids, such as several types of closed circuit television, and considered consumer electronics such as smartphones and tablets, and other low vision aids such as coloured filters and optical prisms, which are commonly prescribed in low-vision rehabilitation as they are supposed to improve reading in some people) commpared to another device or aid	**Primary**: reading speed in words per minute; **secondary**: reading duration and acuity, ease and frequency of use, QOL, AEs	Jan 2018	CENTRAL, MEDLINE Ovid, Embase Ovid, BIREME LILACS, OpenGrey, ISRCTN registry, ClinicalTrials.gov, WHO ICTRP

© 2021 Assi L et al. *JAMA Ophthalmology.*

Systematic Review	Primary Objective	Population	Intervention / Comparison	Outcomes	Date of search	Databases searched
Wang 2017 b	To provide a systematic review of the literature comparing patient-centered and visual quality outcomes between premium IOL options and standard monofocal implants as well as laser-assisted cataract surgery among adult patients undergoing cataract surgery	Adult patients undergoing cataract surgery	Premium IOL options and standard monofocal implants as well as laser-assisted cataract surgery	Dysphotopsias, contrast sensitivity, spectacle independence, QOL, functional outcomes, patient expectations, patient satisfaction, IOL exchange	Sep 2016	PubMed, EMBASE
Xu 2017	To compare the clinical performance between trifocal and bifocal IOLs in bilateral cataract and/or refractive lens exchange surgery	Patients who underwent cataract and/or refractive lens exchange surgery	Bilateral implantation of trifocal IOLs and bifocal IOLs during cataract or refractive lens exchange surgery	**Primary**: best uncorrected distance VA, uncorrected intermediate VA, uncorrected near VA, defocus curve, spectacle independence, patient satisfaction, contrast sensitivity; **secondary**: residual sphere, spherical equivalent, cylinder, complications	Oct 2016	PubMed, EMBASE, Cochrane Controlled Trials Register, Web of Science
Yang 2018	To compare a trifocal IOL and a bifocal IOL implantation in improving visual function after cataract surgery	Patients who underwent cataract surgery with trifocal or bifocal IOL implantation in one or both eyes	Trifocal IOL implantation compared to bifocal IOL implantation in one or both eyes	**Primary**: uncorrected and corrected near, intermediate and distance VA, contrast sensitivity and subjective perception of vision quality; **secondary**: refractive error	NR	EMBASE, PubMed
Zhou 2014	To evaluate the safety and efficacy of anti-VEGF therapy, thus providing high-quality evidence from a large sample for the clinical practice of anti-VEGF therapy in the treatment of macular oedema secondary to CRVO	Patients diagnosed with macular oedema secondary to CRVO	Intravitreal anti-vascular endothelial growth factor compared to sham treatment	**Primary**: changes in BCVA and CRT from baseline; **secondary**: proportion of eyes changing 15 or more letters on the ETDRS chart, the proportion with neovascularization, changes in the 25-item Visual Function Questionnaire	Apr 2013	MEDLINE, CENTRAL, EMBASE

© 2021 Assi L et al. *JAMA Ophthalmology.*

Systematic Review	Primary Objective	Population	Intervention / Comparison	Outcomes	Date of search	Databases searched
Zhu 2016	To assess the effects of anti-VEGF therapy for CNV, compared with other treatments, sham treatment or no treatment, in people with pathological myopia	Patients who had CNV secondary to pathological myopia (with a refractive error of -6.0 dioptres or more and an axial length greater than 26.5 mm)	Anti-VEGF therapy compared to another treatment (e.g. photodynamic therapy with verteporfin, laser photocoagulation, macular surgery, another anti-VEGF), sham treatment or no treatment	**Primary**: Mean change from baseline in BCVA at 1 year after treatment, and proportion of participants with a gain of 3+ lines in BCVA at 1 year after treatment; **secondary**: change in central macular thickness, proportion of participants with CNV angiographic closure, percentage of participants with newly developed chorioretinal atrophy or progression of pre-existing chorioretinal atrophy, vision-related QOL, AEs	Jun 2016	CENTRAL, Ovid MEDLINE, Ovid MEDLINE In-Process and Other Non-Indexed Citations, Ovid MEDLINE Daily, Ovid OLDMEDLINE, Embase

Abbreviations: AEs, adverse events; AMD, age-related macular degeneration; AMED, Allied and Complementary Medicine Database; anti-VEGF, anti-vascular endothelial growth factor; BCVA, best-corrected visual acuity; CENTRAL, Cochrane Central Register of Controlled Trials; CINAHL, Cumulative Index to Nursing and Allied Health Literature; CNV, choroidal neovascularization; CPCIS, Conference Proceedings Citation Index-Science; CPCI-SSH, Conference Proceedings Citation Index–Social Science and Humanities; CRD, Centre for Reviews and Dissemination; CRT, central retinal thickness; ETDRS, Early Treatment in Diabetic Retinopathy Study; HTA, Health Technology Assessment; ICTRP, International Clinical Trials Registry Platform; IOL, intraocular lenses; IOP, intraocular pressure; LILACS, Latin American and Caribbean Health Sciences Literature Database; mRCT, metaRegister of Controlled Trials; NR, not reported; NSAIDs, nonsteroidal anti-inflammatory drugs; OAG, open angle glaucoma; OCT, ocular coherence tomography; PROMs, patient-reported outcome measures; QOL, quality of life; RCTs, randomized controlled trials; RP, retinitis pigmentosa; RVO, retinal vein occlusion; SCI-Expanded, Science Citation Index-Expanded; SCIT, subcutaneous immunotherapy; SLT, selective laser trabeculoplasty; SLIT, sublingual immunotherapy; TED, thyroid eye disease; USPSTF, U.S. Preventive Services Task Force; VA, visual acuity; WHO, World Health Organization; WT, waiting time.

© 2021 Assi L et al. *JAMA Ophthalmology.*

eTable 3. Comparisons of Quality of Life Impact of Different Ophthalmic Interventions by Systematic Review

Systematic Review[1]	Quality of Life Outcome or Measure (reported by SR)	# of participants (# of studies)[2]	Studies Included[2,3]	Countries of primary studies[2]	Results/Findings (as reported by SR)	Quality of the Evidence (rated by SR)
**AGE-RELATED CATARACT**						
**Immediate sequential bilateral cataract surgery (same-day) compared to different date bilateral cataract surgery**						
Kessel 2015 (Denmark)[1]	Subjective satisfaction with visual function (assessed using the VF-7, VF-14)	2096 (2 RCTs)		Finland, Spain	Better VRQOL in the same-day bilateral cataract surgery group than in the different date bilateral cataract surgery group after 1 month of follow-up (standardized MD=0.01, 95% CI=-0.47, 0.48, I^2 = 95%).	Moderate
**Cataract surgery among those with fair compared to poor preoperative visual acuity**						
Kessel 2016 (Denmark)[2]	VRQOL (assessed using the VF-14)	249 (2 trials)		Canada, USA	Similar postoperative VRQOL in patients with fair and poor preoperative visual acuity (MD=-3.01, 95% CI=-10.32, 4.30).	Low
**Cataract extraction and IOL implantation done as day compared to in-patient cases**						
Lawrence 2015 (Bahrain)[3]	VRQOL before and after surgery (measured using VF-14)	305 (1 RCT)		Spain	Similar change in VRQOL scores between the day care vs in-patient groups at four months postoperatively (25.2, SD=21.2 vs 23.5, SD=25.7, *P*=.30).	NR
**Trifocal compared to bifocal IOL implantation**						
Jin 2019 (China)[4]	Spectacle independence (assessed using the NEI-RQL-42, VF-14)	55 (2 RCTs)	Jonker 2015*, Cochener 2016*	NL, France	Similar spectacle independence (assessed using QOL instruments) in both groups (RR=0.89, 95% CI=0.71, 1.12, I^2= 0%).	NR
Xu 2017 (China)[5]	Patient satisfaction (assessed using the NEI-RQL-42, NEI-VFQ-25, VF-14)	49 (2 RCTs)	Cochener 2016*, Gundersen 2016	France, Norway	Better patient satisfaction (assessed using QOL instruments) in the trifocal than in the bifocal group (OR=1.27, 95% CI=0.07, 22.72, I^2=NA).	Moderate
Yang 2018 (China)[6]	Subjective visual quality (assessed using the NEI-RQL-42, VF-14, NEI-VFQ-25, NEI-VFQ-39, Quality of Vision)	263 (3 RCTs, 1 cohort)	Jonker 2015*, Cochener 2016*, Gundersen 2016a*, Gundersen 2016b	France, NL, Norway	No statistically significant difference in VRQOL subgroup scores between both groups (*P*>0.06 in all cases) or in overall VRQOL (estimates not reported).	NR
**Multifocal IOL compared to monofocal IOL implantation in cataract surgery**						
Khandelwal 2019 (USA)[7]	VRQOL (assessed using VF-7, VF-14, satisfaction with vision questionnaires)	596 (6 RCTs)	Cillino 2008*, Leyland 2002, Nijkamp 2004*, Peng 2012, Sen 2004*, Zhao 2010*	Italy, UK, NL, Finland, China	More favorable visual function/QOL outcomes in the multifocal IOLs group (pooled random effects standardized MD=-0.54, 95% CI=-1.12, 0.04, I^2=87.9%).	NR

© 2021 Assi L et al. *JAMA Ophthalmology.*

Systematic Review[1]	Quality of Life Outcome or Measure (reported by SR)	# of participants (# of studies)[2]	Studies Included[2,3]	Countries of primary studies[2]	Results/Findings (as reported by SR)	Quality of the Evidence (rated by SR)
de Silva 2016 (UK)[8]	VRQOL (assessed using VF-7, VF-14)	435 (4 RCTs)	Cillino 2008*, Nijkamp 2004*, Sen 2004*, Zhao 2010*	Italy, NL, Finland, China	Some evidence of more favorable outcomes in the multifocal group, but size of the effect was small and inconsistent (wide CIs). Results were not pooled due to high inconsistency between studies (I^2=92%).	Very low
Wang 2017 b (USA)[9]	VRQOL (assessed using VF-14, VF-7)	NR (3 RCTs)	Nijkamp 2004*, Cillino 2008*, Zhao 2010*	NR	In two studies, VRQOL scores were higher (i.e., better VRQOL) in the multifocal than in the monofocal group (87.1% in the monofocal group vs 93.8%-99.1% in the multifocal groups, p=0.002; and 89.8% in the monofocal group vs 97.3% in the multifocal group, p<0.05). In one study,VRQOL was similar in the monofocal and multifocal groups.	NR
**Multifocal IOL implantation compared to monovision in cataracat surgery**						
Wang 2017 b (USA)[9]	VRQOL (assessed using VF-14)	NR (1 RCT)		NR	Similar overall VRQOL and near and distance vision VRQOL in both groups (estimates not provided).	NR
**Toric IOL implantation compared to limbal relaxing incisions for astigmatism correction during cataract surgery**						
Lake 2019 (Brazil)[10]	VRQOL (assessed using VF-14)	40 (1 RCT)		Spain	Little difference in VRQOL in the toric IOL group compared to the limbal relaxing incisions group (MD=-3.01, 95% CI=-8.56, 2.54, I^2=NA).	Low
**Toric IOL implantation compared to nontoric IOL implantation for astigmatism correction during cataract surgery**						
Wang 2017 b (USA)[9]	VRQOL (assessed using NEI-RQL-42)	NR (1 non-RCT)		NR	Better VRQOL on the dimensions of clarity of vision, distance vision, glare, and satisfaction with correction in the toric IOL group compared to nontoric IOL group (estimates not provided).	NR
**Aspheric IOL implantation compared to spherical IOL during cataract surgery**						
Schuster 2013 (Germany)[11]	VRQOL (assessed using the NEI-VFQ-25, VF-14, ADVS)	NR (6 RCTs)		Europe, Asia, North America	Partly contradictory results. Four studies found similar VRQOL in aspheric and spherical IOL implantation, while two studies reported statistically significant improvements for the aspheric group (estimates not provided).	NR
**Topical NSAID plus steroids compared to topical steroids alone after cataract surgery**						
Lim 2016 (UK)[12]	QOL (assessed using the Comparison of Ophthalmic Medications for Tolerability questionnaire)	74 (1 RCT)		Canada	Similar QOL in both groups (estimates not provided).	NR

© 2021 Assi L et al. *JAMA Ophthalmology.*

Systematic Review[1]	Quality of Life Outcome or Measure (reported by SR)	# of participants (# of studies)[2]	Studies Included[2,3]	Countries of primary studies[2]	Results/Findings (as reported by SR)	Quality of the Evidence (rated by SR)
**CORNEAL CONDITIONS**						
**Antibiotic-only group (gatifloxacin and placebo) compared to antibiotic-steroid group (gatifloxacin and 0.1% dexamethasone) for bacterial keratitis**						
Herretes 2014 (USA)[13]	VRQOL (assessed using VF-14)	26 (1 RCT)		Canada	No difference in VRQOL scores between both groups (6.2 for the antibiotic-only group vs 9.7 for the antibiotic-steroid group, SDs not available, *P*=0.42).	NR
**AGE-RELATED MACULAR DEGENERATION**						
**Bevacizumab compared to ranibizumab for neovascular age-related macular degeneration**						
Solomon 2019 (USA)[14]	HRQOL (assessed using the EQ-5D)	548 (1 RCT)	IVAN 2013*	UK	Similar HRQOL summary score in the bevacizumab compared to the ranibizumab group at one year follow-up (median score=0.85, IQR=0.73, 1.00, in both groups).	Moderate
Low 2019 (USA)[15]	HRQOL (assessed using HRQOL and macular degeneration-related QOL, specific tools NR)	NR (1 RCT)	Chakravarthy 2015 (IVAN 2013)*	NR	No between-group differences for either general or macular degeneration-related QOL in the bevacizumab compared to the ranibizumab group (estimates not provided).	NR
**As needed compared to monthly injections for administration of anti-VEGF agents for neovascular AMD**						
Li 2020 (Italy)[16]	QOL (assessed using the EQ-5D, Macular Disease Dependent QOL, Macular Disease Treatment Satisfaction Questionnaires)	498 (1 RCT)		UK	No significant difference in QOL between monthly and as needed injections at 1 year (similar median scores, but estimates not provided).	Low
**Extended-fixed (such as injections every 2 or 3 months) compared to monthly injections for administration of anti-VEGF agents for neovascular AMD**						
Li 2020 (Italy)[16]	VRQOL (assessed using the NEI-VFQ-25)	1220 (1 RCT)			No significant difference in QOL between extended fixed and monthly injections at 1 year (MD=-0.59, 95% CI=-2.22, 1.04).	Moderate
**Photocoagulation compared to submacular surgery for subfoveal neovascular AMD**						
Virgili 2007 (Italy)[17]	HRQOL (assessed using the SF-36)	70 (1 RCT)		USA	No statistically significant differences in HRQOL between the photocoagulation and submacular surgery groups (RR for loss of 5+ points of the SF-36 Physical Component Score=0.82, 95% CI=0.32, 2.10; RR for loss of 5+ points of the SF-36 Mental Component Score=0.96, 95% CI=0.32, 2.90).	NR
**Macular translocation compared to photodymanic therapy for neovascular AMD**						
Eandi 2008 (Italy)[18]	VRQOL subscales (assessed using the NEI-VFQ-25 subscales)	50 (1 RCT)		Germany	Some VRQOL subscales favored macular translocation over photodymanic therapy (MD in general vision score=9.9, 95% CI=1.54, 18.26; and and MD in mental health score=12.6, 95% CI=1.06, 24.14), but the overall score was not available.	NR

© 2021 Assi L et al. *JAMA Ophthalmology.*

Systematic Review[1]	Quality of Life Outcome or Measure (reported by SR)	# of participants (# of studies)[2]	Studies Included[2,3]	Countries of primary studies[2]	Results/Findings (as reported by SR)	Quality of the Evidence (rated by SR)
**RETINA (OTHER)**						
**Anti-VEGF therapy compared to control group (not specified) for diabetic macular edema**						
Ollendorf 2013 (USA)[19]	General health status/HRQOL (assessed using EQ-5D)	NR (2 RCTs)	NR	NR	No significant differences in HRQOL between the treatment and control groups (estimates not provided).	NR
Ollendorf 2013 (USA)[19]	VRQOL (assessed using NEI-VFQ-25)	NR (2 RCTs)	NR	NR	Improved VRQOL in the treatment group, primarily limited to vision-related domains (estimates not provided).	NR
**Macular hole surgery with internal limiting membrane peeling compared to without**						
Spiteri 2013 (UK)[20]	HRQOL (assessed using the EQ-5D), and VRQOL (assessed using the NEI-VFQ-25)	NR (1 RCT)		NR	Similar VRQOL and HRQOL in both groups (*P*=0.97, estimates not provided).	NA
**GLAUCOMA**						
**Initial medical treatment compared to initial trabeculectomy for open angle glaucoma**						
Burr 2012 (UK)[21]	HRQOL (asssessed using the Visual Activities Questionnaire, Glaucoma Health Perceptions Index, Sickness Impact Profile, Centre for Epidemiological studies - Depression Score, Health Perceptions Index)	607 (1 RCT)		US	No differences in overall visual function, systemic symptoms, and overall well being between groups, however, the initial trabeculectomy group had worse QOL outcomes related to visual taks and eye discomfort (estimates not provided).	NA
**LOW VISION**						
**Optical devices: Hand-held electronic device plus optical device compared to optical device for adults with low vision**						
Virgili 2018 b (Italy)[22]	VRQOL/estimated perceived difficulty (assessed using the NV-VFQ-25)	100 (1 RCT)	Taylor 2017	UK	Better VRQOL / less perceived difficulty at 2 months among adults who used hand-held electronic devices compared to optical devices alone (MD=0.57, 95% CI=0.33, 0.81).	Moderate
**Optical devices: Prism spectacles compared to conventional spectacles for adults with low vision**						
Virgili 2018 b (Italy)[22]	VRQOL (assessed using the NEI-VFQ-25)	153 (1 RCT)	Smith 2005	UK	Similar VRQOL among adults who used custom or standard prism spectacles compared to conventional spectacles at 3 months (MD=0, 95% CI=-5.62, 5.62).	Moderate
**Optical devices: Stand-mounted CCTV with visual rehabilitation compared to visual rehabilitation alone for adults with low vision**						
Virgili 2018 b (Italy)[22]	VRQOL (assessed using IVI, Activity Inventory)	31 (1 RCT)	Jackson 2017	USA	No statistically significant difference between the 2 groups for any domain (emotional, mobility, reading) at 1 month after visual rehabitilation consultation (estimates not provided).	Low

© 2021 Assi L et al. *JAMA Ophthalmology.*

Systematic Review[1]	Quality of Life Outcome or Measure (reported by SR)	# of participants (# of studies)[2]	Studies Included[2,3]	Countries of primary studies[2]	Results/Findings (as reported by SR)	Quality of the Evidence (rated by SR)
**Vision rehabitilation: Methods of enhancing vision (e.g., customised prism glasses) compared to active control for vision impaired adults**						
van Nispen 2020 (NL)[23]	HRQOL (assessed using the EQ-5D, SF-36)	443 (2 RCTs)	Burggraaff 2012, Stelmack 2017	NL, USA	Modest and imprecise overall effects on HRQOL (standardized MD=-0.09, 95% CI=-0.28. 0.09, I^2=0%).	Very low
van Nispen 2020 (NL)[23]	VRQOL (assessed using the VISQOL, LVQOL subscales, VA-LV-VFQ, VFQ-25, Activity Inventory, IVI)	660 (7 RCTs)	Burggraaff 2012*, Stelmack 2017*, Pearce 2011, Draper 2016, Jackson 2017, Leat 2017, Taylor 2017	Canada, NL, UK, USA	Small, but significant VRQOL benefits in the groups receiving vision rehabitilation (standardized MD=-0.24, 95% CI=-0.40, -0.08; I^2=3%).	Moderate
**Vision rehabitilation: Multidisciplinary rehabilitation (e.g., low vision rehabitilation plus home visit) compared to active control for vision impaired adults**						
van Nispen 2020 (NL)[23]	HRQOL (assessed using the WHO-QOL, SF-36)	375 (2 RCTs)	Christy 2012*, Reeves 2004*	India, UK	Mutidisciplinary rehabilitation was associated with small or no benefit in HRQOL compared to active control (standardized MD=-0.10, 95% CI=-0.31, 0.12; I^2=0%).	Very low
van Nispen 2020 (NL)[23]	VRQOL (assessed using the IVI, FAQ, VCM1)	464 (3 RCTs)	Christy 2012*, Reeves 2004*, McCabe 2000	India, UK, USA	Overall multidisciplinary rehabilitation was not associated with benefits in VRQOL compared to active control (standardized MD=0.01, 95% CI=-0.18, 0.20; I^2 = 0%).	Low
**Enhanced/multidisciplinary low vision rehabitilation services compared to conventional low vision rehabitilation or optometric services**						
Rees 2010 (Australia)[24]	QOL (assessed using the VCM1, LVQOL, SF-36)	535 (2 trials)	Reeves 2004*, De Boer 2006,	NL, UK, Australia	In the 2 trials identified (highest quality evidence), no differences in QOL were noted between the different rehabitilation services (estimates not provided).	NR
**VISION SCREENING**						
**Vision screening (visual acuity test) compared to vision screening (question about vision) as part of a multi-component screening package (standard of care) among older adults**						
Clarke 2018 (UK)[25]	VRQOL (assessed using the NEI-VFQ-25)	1807 (1 RCT)	Smeeth 2003	UK	Little evidence of any difference in VRQOL (MD=0.4, 95% CI=−1.7, 2.5, *P*=0.69).	High
Chou 2016 (USA)[26]	VRQOL (assessed using the NEI-VFQ-25)	3346 (1 RCT)	Smeeth 2003	UK	Similar VRQOL in both groups (MD=0.4, 95% CI=−1.7, 2.5).	Fair
**Rural refractionist compared to university optometrist in school vision screenings for correctable visual acuity deficits**						
Evans 2018 (UK)[27]	VRQOL (assessed using the NEI- RQL-42)	198 (1 RCT)	WEAR 2017	China	Little evidence of any important differences in VRQOL between the two groups at two months (MD=1.81, 95% CI=-1.01, 4.63).	NR
**Self-refraction compared to university optometrist in school vision screenings for correctable visual acuity deficits**						
Evans 2018 (UK)[27]	VRQOL (assessed using the NEI-RQL-42)	188 (1 RCT)	WEAR 2017	China	Little evidence of any important differences in VRQOL between the groups at 2 months (MD=0.82, 95% CI=-2.00, 3.64).	NR

© 2021 Assi L et al. *JAMA Ophthalmology.*

Systematic Review[1]	Quality of Life Outcome or Measure (reported by SR)	# of participants (# of studies)[2]	Studies Included[2,3]	Countries of primary studies[2]	Results/Findings (as reported by SR)	Quality of the Evidence (rated by SR)
**AMBLYOPIA**						
**Conventional occlusion compared to atropine penalization for amblyopia**						
Li 2019 (USA)[28]	Psychosocial impact and QOL (assessed using patient questionnaires, Amblyopia Treatment Index)	256 (2 trials)		India, USA	Similar QOL results in both groups. Social stigma and adherence measures favored atropine over conventional occlusion.	Moderate
**UVEITIS**						
**Fluocinolone acetonide intravitreal implant compared to standard of care systemic management in chronic non-infections uveitis**						
Brady 2016 (USA)[29]	VRQOL (assessed using NEI-VFQ-25)	235 (1 RCT)		Australia, UK, USA	Significantly greater improvement in VRQOL in the fluocinolone acetonide intravitreal implant group compared to standard of care systemic management at 12 months (MD=7.29, 95% CI=3.11, 11.42) and 24 months (MD=4.64, 95% CI= 0.14, 9.15).	Moderate
**TRICHIASIS**						
**Posterior lamellar tarsal rotation surgery compared to epilation for minor trichiasis**						
Burton 2015 (UK)[30]	VRQOL	1300 (1 RCT)		Ethiopia	Better VRQOL in the surgery group at 12 months follow-up (78% vs 33% reported better subjective improvement in vision, difference was statistically significant).	NR

Abbreviations: ADVS, Activities of Daily Vision Scale; Anti-VEGF, anti-vascular endothelial growth factor; AMD, age-related macular degeneration; EQ-5D, EuroQol- 5 Dimension; FAQ, Functional Assessment Questionnaire; HRQOL, health-related quality of life; IQR, interquartile range; IVI, impact of vision impairment; LVQOL, low vision quality of life questionnaire; MD, mean difference; NEI-RQL-42, National Eye Institute Refractive Error Quality of Life Instrument-42; NEI-VFQ-25, National Eye Institute 25-Item Visual Function Questionnaire; QOL, quality of life; RCT, randomized-controlled trial; SD, standard deviation; SF-36, 36-Item Short Form Health Survey; UK, United Kingdom; USA, United States of America; VA-LV-VFQ, Veterans Affairs Low Vision Visual Functioning Questionnaire; VCM1, Vision-related quality of life Core Measure 1; VFQ-25, 25-Item Visual Function Questionnaire; VISQOL, vision-related quality of life; VF-7, Visual Function Index; VF-14, Visual Function Index; VRQOL, vision-related quality of life; WHO-QOL, World Health Organization Quality of Life.

© 2021 Assi L et al. *JAMA Ophthalmology.*

eTable 4. Studies Included in Table 2

Study	Overlapping studies included
**Age-related cataract**	
Chou, et al	No overlapping studies with other reviews
Hodge, et al	Harwood 2005, Laidlaw 1998
Conner-Spady, et al	Harwood 2005, Laidlaw 1998
Casparis, et al	No overlapping studies with other reviews
Ishikawa, et al	Castells 1999, Laidlaw 1998, Foss 2006, Javitt 1993, Elliot 1997, Elliot 2000, Castells 2006
Frampton, et al	Foss 2006, Castells 2006
Ishikawa, et al	Castells 1999, Rasanen 2006, Laidlaw 1998, Foss 2006, Elliot 2000, Castells 2006
Frampton, et al	Foss 2006, Laidlaw 1998, Castells 2006
Riaz, et al	No overlapping studies with other reviews
**Refractive error**	
Chou, et al	No overlapping studies with other reviews
**Age-related macular degeneration**	
Chou, et al	PIER, MARINA, VISION
Solomon, et al	ANCHOR 2006, MARINA 2006
Sarwar, et al	VIEW 1, VIEW 2
Giansanti, et al	No overlapping studies with other reviews
Evans, et al	No overlapping studies with other reviews
Evans, et al	No overlapping studies with other reviews
Evans, et al	Ma 2012
Liu, et al	Piermarocchi 2012, Ma 2012
**Retina (other)**	
Virgili, et al	No overlapping studies with other reviews
Braithwaite, et al	COPERNICUS 2012, CRUISE 2010, GALILEO 2013
Zhou, et al	COPERNICUS, CRUISE, GALILEO
Ford, et al	COPERNICUS, GALILEO, CRUISE
Mitry, et al	No overlapping studies with other reviews
Zhu, et al	No overlapping studies with other reviews
Lescrauwaet, et al	No overlapping studies with other reviews
Neffendorf, et al	No overlapping studies with other reviews
Brito-García, et al	No overlapping studies with other reviews
**Glaucoma**	
Rolim de Moura, et al	No overlapping studies with other reviews

© 2021 Assi L et al. *JAMA Ophthalmology.*

Chi, et al	No overlapping studies with other reviews
**Low vision**	
van Nispen, et al	No overlapping studies with other reviews
van Nispen, et al	Stelmack 2008, Acton 2016
van Nispen, et al	Stelmack 2008, Acton 2016
**Vision screening**	
Evans, et al	No overlapping studies with other reviews
**Rhinoconjunctivitis**	
Erekosima, et al	Frew 2006, Tabar 2008, Ferrer 2005, Walker 2001
Kim, et al	Cantani 1997, Kuna 2011
Kim, et al	de Bot 2012, Roder 2007
Lin, et al	Moreno-Ancillo 2007, de Bot 2012, Röder 2007, O’Hehir 2009, Okubo 2008, Makino 2010, Fujimura 2011, Di Rienzo 2006
Rodrigo, et al	Fokkens 2007, Kaiser 2007, Martin 2007, Andrews 2009, Jacobs 2009
Rodrigo, et al	Maspero 2008, Patel 2008, GlaxoSmithKline FFR100652
**Uveitis**	
Urruticoechea-Arana, et al	VISUAL I, VISUAL II
Squires, et al	VISUAL I, VISUAL II, HURON
Squires, et al	VISUAL I, VISUAL II
**Trichiasis**	
Burton, et al	No overlapping studies with other reviews
**Thyroid eye disease or Graves’ ophthalmopathy**	
Viani, et al	Prummel 2004
Rajendram, et al	Prummel 2004

© 2021 Assi L et al. *JAMA Ophthalmology.*

eReferences.

1. Kessel L, Andresen J, Erngaard D, Flesner P, Tendal B, Hjortdal J. Immediate Sequential Bilateral Cataract Surgery: A Systematic Review and Meta-Analysis. *Journal of ophthalmology.* 2015;2015:912481.
2. Kessel L, Andresen J, Erngaard D, Flesner P, Tendal B, Hjortdal J. Indication for cataract surgery. Do we have evidence of who will benefit from surgery? A systematic review and meta-analysis. *Acta ophthalmologica.* 2016;94(1):10-20.
3. Lawrence D, Fedorowicz Z, van ZE. Day care versus in-patient surgery for age-related cataract. 2015(11). doi:10.1002/14651858.CD004242.pub5.
4. Jin S, Friedman DS, Cao K, et al. Comparison of postoperative visual performance between bifocal and trifocal intraocular Lens based on randomized controlled trails: a meta-analysis. *BMC ophthalmology.* 2019;19(1):78.
5. Xu Z, Cao D, Chen X, Wu S, Wang X, Wu Q. Comparison of clinical performance between trifocal and bifocal intraocular lenses: A meta-analysis. *PloS one.* 2017;12(10):e0186522.
6. Yang J-J, Liu Q-P, Li J-M, Qin L. Comparison of visual outcomes with implantation of trifocal versus bifocal intraocular lens after phacoemulsification: a Meta-analysis. *International journal of ophthalmology.* 2018;11(3):484-492.
7. Khandelwal SS, Jun JJ, Mak S, Booth MS, Shekelle PG. Effectiveness of multifocal and monofocal intraocular lenses for cataract surgery and lens replacement: a systematic review and meta-analysis. *Graefe's archive for clinical and experimental ophthalmology = Albrecht von Graefes Archiv fur klinische und experimentelle Ophthalmologie.* 2019;257(5):863-875.
8. de Silva SR, Evans JR, Kirthi V, Ziaei M, Leyland M. Multifocal versus monofocal intraocular lenses after cataract extraction. *Cochrane Database of Systematic Reviews.* 2016;2016(12).
9. Wang SY, Stem MS, Oren G, Shtein R, Lichter PR. Patient-centered and visual quality outcomes of premium cataract surgery: a systematic review. *European journal of ophthalmology.* 2017;27(4):387-401.
10. Lake JC, Victor G, Clare G, Porfirio GJ, Kernohan A, Evans JR. Toric intraocular lens versus limbal relaxing incisions for corneal astigmatism after phacoemulsification. *The Cochrane database of systematic reviews.* 2019;12:CD012801.
11. Schuster AK, Tesarz J, Vossmerbaeumer U. The impact on vision of aspheric to spherical monofocal intraocular lenses in cataract surgery: a systematic review with meta-analysis. *Ophthalmology.* 2013;120(11):2166-2175.
12. Lim BX, Lim CH, Lim DK, Evans JR, Bunce C, Wormald R. Prophylactic non-steroidal anti-inflammatory drugs for the prevention of macular oedema after cataract surgery. *The Cochrane database of systematic reviews.* 2016;11:CD006683.
13. Herretes S, Wang X, Reyes JMG. Topical corticosteroids as adjunctive therapy for bacterial keratitis. *The Cochrane database of systematic reviews.* 2014(10):CD005430.
14. Solomon SD, Lindsley K, Vedula SS, Krzystolik MG, Hawkins BS. Anti-vascular endothelial growth factor for neovascular age-related macular degeneration. *The Cochrane database of systematic reviews.* 2019;3:CD005139.
15. Low A, Faridi A, Bhavsar KV, et al. Comparative effectiveness and harms of intravitreal antivascular endothelial growth factor agents for three retinal conditions: a systematic review and meta-analysis. *The British journal of ophthalmology.* 2019;103(4):442-451.
16. Li E, Donati S, Lindsley KB, Krzystolik MG, Virgili G. Treatment regimens for administration of anti-vascular endothelial growth factor agents for neovascular age-related macular degeneration. *Cochrane Database of Systematic Reviews.* 2020(5).
17. Virgili G, Bini A. Laser photocoagulation for neovascular age-related macular degeneration. 2007(3). doi:10.1002/14651858.CD004763.pub2.
18. Eandi C, Giansanti F, Virgili G. Macular translocation for neovascular age-related macular degeneration. 2008(4). doi:10.1002/14651858.CD006928.pub2.
19. Ollendorf DA, Colby JA, Pearson SD. Comparative effectiveness of anti-vegf agents for diabetic macular edema. *International Journal of Technology Assessment in Health Care.* 2013;29(4):392-401.
20. Spiteri CK, Lois N, Scott N, et al. Vitrectomy with internal limiting membrane (ILM) peeling versus vitrectomy with no peeling for idiopathic full-thickness macular hole (FTMH). 2013(6). doi:10.1002/14651858.CD009306.pub2.
21. Burr J, Azuara-Blanco A, Avenell A, Tuulonen A. Medical versus surgical interventions for open angle glaucoma. 2012(9). doi:10.1002/14651858.CD004399.pub3.
22. Virgili G, Acosta R, Bentley SA, Giacomelli G, Allcock C, Evans JR. Reading aids for adults with low vision. *The Cochrane database of systematic reviews.* 2018;4:CD003303.
23. van Nispen RM, Virgili G, Hoeben M, et al. Low vision rehabilitation for better quality of life in visually impaired adults. *The Cochrane database of systematic reviews.* 2020;1:CD006543.
24. Rees G, Ponczek E, Hassell J, Keeffe JE, Lamoureux EL. Psychological outcomes following interventions for people with low vision: A systematic review. *Expert Review of Ophthalmology.* 2010;5(3):385-403.
25. Clarke EL, Evans JR, Smeeth L. Community screening for visual impairment in older people. *The Cochrane database of systematic reviews.* 2018;2:CD001054.
26. Chou R, Dana T, Bougatsos C, Grusing S, Blazina I. Screening for Impaired Visual Acuity in Older Adults: Updated Evidence Report and Systematic Review for the US Preventive Services Task Force. *JAMA.* 2016;315(9):915-933.
27. Evans, Jr., Morjaria P, Powell C. Vision screening for correctable visual acuity deficits in school-age children and adolescents. 2018(2). doi:10.1002/14651858.CD005023.pub3.
28. Li T, Qureshi R, Taylor K. Conventional occlusion versus pharmacologic penalization for amblyopia. *The Cochrane database of systematic reviews.* 2019;8:CD006460.
29. Brady C, Villanti A, Law H, et al. Corticosteroid implants for chronic non-infectious uveitis. 2016(2). doi:10.1002/14651858.CD010469.pub2.

© 2021 Assi L et al. *JAMA Ophthalmology.*

30. Burton M, Habtamu E, Ho D, Gower EW. Interventions for trachoma trichiasis. *Cochrane Database of Systematic Reviews.* 2015;2015(11).

© 2021 Assi L et al. *JAMA Ophthalmology.*
